# Overcoming Drug Resistance by Paclitaxel Resistance in Triple‐Negative Breast Cancer

**DOI:** 10.1002/advs.77249

**Published:** 2026-08-20

**Authors:** Bo Chen, Yangyi Wang, Tingting Li, Changqing Guo, Zhe Xie, Zhixian Zhang, Xinghua Han, Wei Wang, Jian Wang, Xiaopeng Ma, Yuanzeng Min

**Affiliations:** ^1^ Department of General Surgery Department of Medical Oncology Department of Neurology The First Affiliated Hospital of USTC Division of Life Sciences and Medicine University of Science and Technology of China Hefei China; ^2^ Department of Bio‐X Interdisciplinary Science at Hefei National Laboratory (HFNL) for Physical Science at the Microscale University of Science and Technology of China Hefei China; ^3^ Department of Clinical Laboratory The First Affiliated Hospital of Anhui Medical University Hefei China; ^4^ School of Chemistry and Materials Science University of Science and Technology of China Hefei China; ^5^ State Key Laboratory of Immune Response and Immunotherapy, Center for Advanced Interdisciplinary Science & Biomedicine of IHM, Division of Life Sciences and Medicine University of Science and Technology of China Hefei China

**Keywords:** chemoresistance, immune checkpoint inhibitors, nanovaccine, neoantigens, resistance to immune checkpoint inhibitors, triple‐negative breast cancer, tumor neoantigen burden

## Abstract

Drug resistance in solid tumors has emerged as a critical challenge, with limited effective therapeutic options available. In this study, we find that chemotherapy induces an increased tumor neoantigens burden (TNB) in breast cancer patients and both non‐resistant and chemo‐resistant triple‐negative breast cancer 4T1 tumor‐bearing mice. Consistently, proteomic analysis reveals that tumor antigens derived from in vitro chemotherapy‐treated paclitaxel (PTX)‐resistant 4T1 cells (chemo‐rAg) are enriched in damage‐associated molecular patterns and neoantigens, highlighting their potential to elicit antitumor immunity against chemo‐resistant tumors. Based on these findings, we utilize the feature of PTX resistance and develop a nanovaccine incorporating chemo‐rAg and a prodrug of a toll‐like receptor 7 (TLR7) agonist. In preclinical mouse models, the nanovaccine effectively suppresses PTX‐resistant 4T1 tumor growth, and it also reverses the innate resistance of immune checkpoint inhibitors. Notably, the therapeutic benefit of the nanovaccine is augmented by chemotherapy, which facilitates in situ release of tumor antigens. These results suggest that a chemo‐rAg‐based nanovaccine represents a promising strategy based on the ′like cures like' principle to overcome drug resistance by utilizing the characteristic of the drug resistance itself.

## Graphical abstract

In the murine triple‐negative breast cancer (TNBC) model, chemotherapy effectively increases tumor neoantigen burden (TNB). Here, the study constructs a liposomal nanovaccine using antigens derived from in vitro chemotherapy‐treated paclitaxel‐resistant TNBC 4T1 cells. The combination of the nanovaccine and immune checkpoint inhibitor (ICI) potently suppresses the growth of subcutaneously implanted paclitaxel‐resistant 4T1 tumors, effectively overcoming the drug resistance.

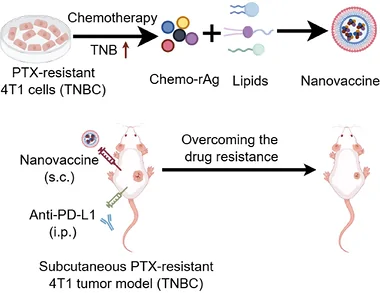

## 1 Introduction

Breast cancer (BC) is ranking on the top cancer incidences among women, while the estimated deaths of BC account for 15% of all cancer death [1]. Triple‐negative breast cancer (TNBC) is the most aggressive and lethal subtype of BC and is more common in BRCA1‐mutated breast cancers [2, 3]. TNBC subtype tends to metastasis or even recurrence, thus resulting in a poor prognosis and worst survival outcome with a median overall survival (OS) of less than two years [4, 5].

In general, whether early‐stage of TNBC or advanced‐stage of TNBC, or even metastatic TNBC (mTNBC), their treatments all relied on chemotherapy during the past twenty years [6]. With the development of omics technology, transcriptomic and genomic studies make the molecular classification of TNBC more specific, which would make the relative treatment more precise [7]. Many targeted therapies to different pathways are developed, which achieve personalized treatments [8]. However, whether it's chemotherapy or targeted therapy, drug resistance during the treatment process has always been a problem and challenge, which is due to the heterogeneity of TNBC [9, 10, 11, 12]. The mTNBCs have also shown strong resistance to chemotherapy, which would affect the drug efficacy and result in a poor prognosis post treatment [13, 14, 15].

On the other side, cancer immunotherapies, which reactivate the patients’ immune system to kill cancer cells, can produce durable response rates (RRs) [16]. Immune checkpoint inhibitors (ICIs) that inhibit negative immune regulation have achieved great success and were approved for clinic use, for example, antibodies against programmed death‐1/programmed death ligand‐1 (anti‐PD‐1 or anti‐PD‐L1) [17]. However, many patients showed a lack of initial response to ICIs’ treatments, whose tumors have less PD‐L1 expression, low levels of cytotoxic T lymphocytes (CTLs) infiltration, and lessened tumor mutational burden (TMB) [17, 18, 19]. Specifically, less tumor neoantigen burden (TNB), including loss of neoantigen expression and defective neoantigen presentation, will undermine the responses to ICIs [20, 21]. The suppressive tumor microenvironment (TME), including increased ratio of regulatory T cells (Tregs) to CTLs, myeloid‐derived suppressor cells (MDSCs), tumor‐associated M2 phenotype macrophages etc., will also fade the responses and cause innate resistance [15, 22].

Monotherapy of pembrolizumab, an anti‐PD‐1 therapy, or atezolizumab, an anti‐PD‐L1 therapy, generated few responders having durable responses [23, 24]. Monotherapy of pembrolizumab failed to significantly improve OS of patients with previously treated mTNBC when compared to chemotherapy only [25]. Higher RRs were observed with atezolizumab in combination with nanoparticle albumin‐bound paclitaxel (nab‐PTX) in patients with PD‐L1^+^ unresectable advanced or mTNBC [26, 27]. Subsequently, pembrolizumab was in combination with PTX, nab‐PTX, or gemcitabine (GEM) plus carboplatin (CBP) and produced significant and clinically meaningful improvement in patients with mTNBC [28, 29, 30]. However, ICI (nivolumab) in combination with chemotherapy doesn't always generate enhanced or significant responses, but sometimes even induces decreased responses. For example, cyclophosphamide (CTX) attenuates the response of nivolumab to 8% in patients with mTNBC, in comparison to 20% for nivolumab only [31]. Systemic administration of carmustine in combination with anti‐PD‐1 treatment results in severe lymphodepletion and depletion of tumor infiltrating lymphocytes (TILs) in mice study [32]. Even more, chemotherapy could induce T cell exhaustion [33, 34]. In summary, ICI therapies in combination with chemotherapy showed either enhanced efficacy or decreased responses in different scenarios, which are difficult to predict or control. However, in fact, more than 80% of active clinic trials of anti‐PD‐1/PD‐L1 are combination therapies by 2020 [35, 36, 37]. So, this kind of strategy of their combinations, which enhance the immune responses and overcome innate resistance, needs to be improved [38].

Together, as demonstrated above, effective strategies to overcome drug resistance, including both chemoresistance and innate resistance to ICIs, in TNBC remain limited. Notably, accumulating evidence indicates that chemotherapy can promote immunogenic cell death (ICD), increase TMB or TNB, thereby sensitizing tumors to ICI therapy [38, 39, 40, 41, 42, 43]. However, there are few studies regarding the immunogenicity of chemo‐resistant tumors. Previously, we developed antigen capturing nanoparticles (ACNPs) to capture neoantigens released from irradiated primary tumor and enhance antigen presentation, thereby improving cancer immunotherapy and the abscopal effect [44]. Subsequently, radiotherapy‐induced neoantigens were applied to study of cancer vaccine [45]. These studies prompted us to hypothesize that antigens derived from chemo‐resistant TNBC, particularly those generated under chemotherapy‐induced selective pressure, could be repurposed as a vaccine source to boost antitumor immunity and potentially overcome drug resistance.

To test this concept, we performed whole‐exome sequencing (WES) and RNA sequencing (RNA‐Seq) on clinical BC patient tumor samples, including 1 patient with TNBC, collected before and after chemotherapy in clinic (Figure 1). The results demonstrated that chemotherapy led to a significant change of neoantigens landscape and a marked increase in TNB, which indicated an enhanced tumor immunogenicity. Similar trends were observed in the non‐resistant murine TNBC 4T1 tumor model. Encouragingly, in the chemo‐resistant TNBC 4T1 tumor model, chemotherapy also showed partly elevated TNB. Although higher chemotherapy doses might be required to increase TNB in this setting, dose escalation is constrained by host toxicity in vivo. So, to avoid this high dose toxicity, we extracted antigens from in vitro treated resistant cells. Proteomic analysis revealed that tumor antigens derived from in vitro chemotherapy‐treated PTX‐resistant 4T1 cells (chemo‐rAg) contained more abundant damage associated molecular patterns (DAMPs), tumor associated antigens (TAAs) and neoantigens compared to untreated PTX‐resistant 4T1 cells (rAg). To promote antigen presentation and construct a multivalent cancer vaccine, chemo‐rAg was co‐formulated with chol‐loxo, a previously reported acid‐responsive toll‐like receptors 7 (TLR7) agonist prodrug (Figure S1), into liposomes [46]. The resulting nanovaccine suppressed the growth of PTX‐resistant 4T1 tumors and prolonged survival in treated mice. Combination therapy with anti‐PD‐L1 further enhanced the therapeutic efficacy. Moreover, in vivo PTX administration triggered in situ release of tumor antigens, which further improved the therapeutic outcomes. Mechanism analyses showed that the nanovaccine enhanced intratumoral T‐cell infiltration and alleviated the immunosuppressive TME. Collectively, these findings may provide valuable insights into the development of novel therapeutic strategies based on the ′like cures like' principle to overcome drug resistance by utilizing the characteristic of drug resistance itself.

**FIGURE 1:**
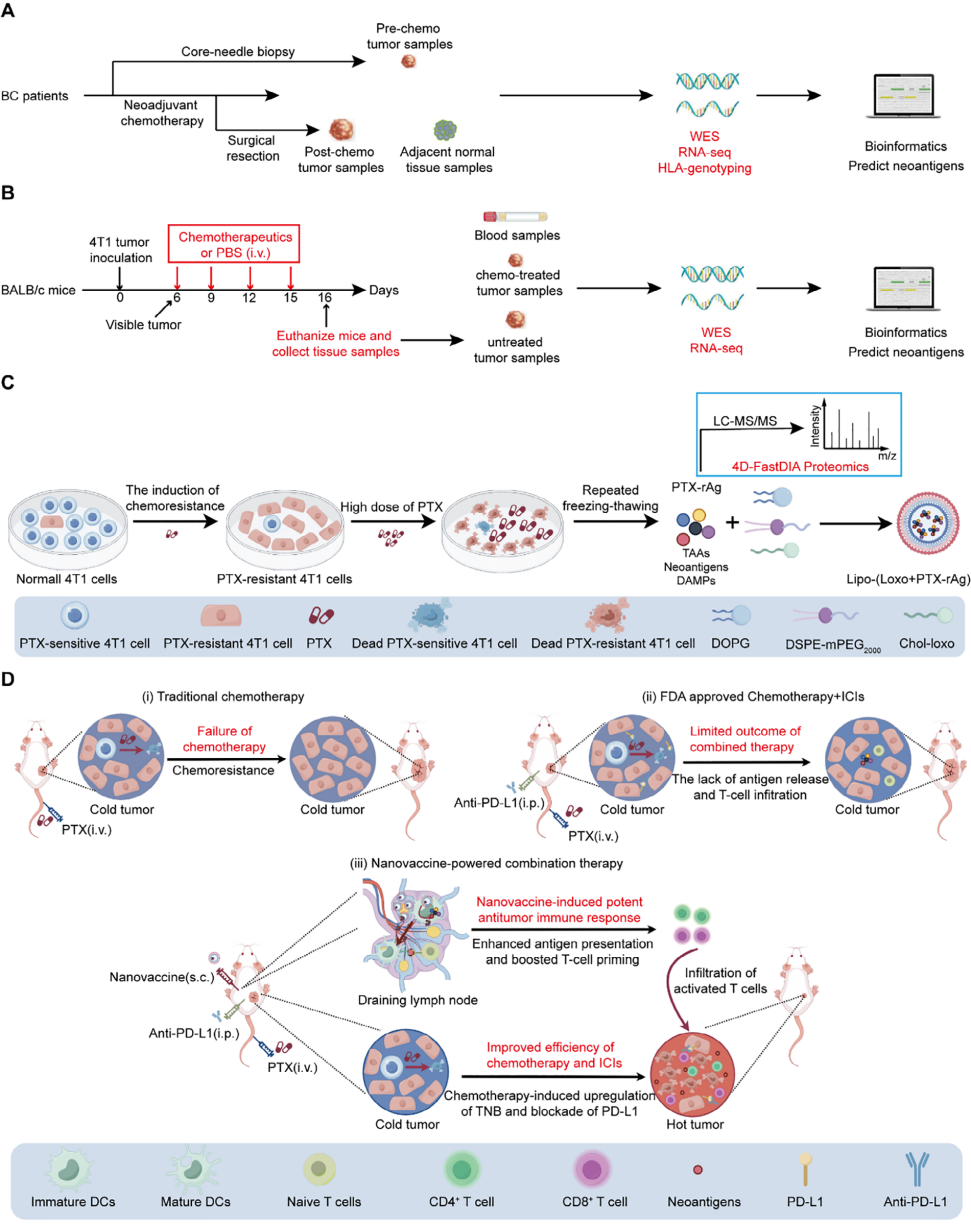
Chemotherapy‐derived nanovaccine to overcome chemoresistance in 4T1 tumors. (A) Schematic illustration showing the process of neoantigens prediction in clinical BC patients. (B) Schematic illustration showing the process of neoantigen prediction in 4T1 tumor‐bearing mice. (C) Schematic illustration showing the proteomics analysis of the tumor antigen samples and the preparation of the nanovaccine Lipo‐(Loxo+PTX‐rAg). (D) Proposed mechanism of the nanovaccine in inhibiting the growth of PTX‐resistant 4T1 tumors in mice. (i) Before immune checkpoint inhibitors (ICIs), chemotherapy was the standard clinical treatment for BC, such as PTX. (ii) Despite FDA approval of combining chemotherapy and ICIs for clinical BC patients, chemoresistance limited the efficacy of this combination therapy in PTX‐resistant 4T1 tumors. (iii) Nanovaccine‐powered combined therapy with chemotherapy and ICIs. The nanovaccine activated potent antitumor T‐cell responses through enhanced DC maturation and antigen presentation. Meanwhile, chemotherapy‐induced in situ tumor antigen release and TNB upregulation, combined with PD‐L1 blockade by ICIs, helps overcome the chemoresistance.

## 2 Results and Discussion

### 2.1 Chemotherapy Upregulated Neoantigens’ Abundance in PTX Resistant Murine 4T1 TNBC Model and the Formulation of the Nanovaccine

#### 2.1.1 Chemotherapy Reshapes Neoantigen Landscape in BC Patients and in PTX‐Resistant Murine 4T1 TNBC Model

Neoadjuvant chemotherapy followed by surgical resection is a standard of care for the purpose of shrinking the tumor and increasing the chance of curative surgery. Previous studies have shown that multiple chemotherapeutic agents, including platinum‐based compounds and temozolomide, can effectively increase TMB in metastatic tumors including BC, thereby it might potentially promote the generation of neoantigens [43]. To verify this effect in the setting of real‐world early or locally advanced BC, tumor samples before and after neoadjuvant chemotherapy, as well as normal tissue samples, were collected from 8 patients with all major BC subtypes (HR^+^, HER2^+^, and TNBC) and subjected to bioinformatics analysis (Data S1). Note that one patient had TNBC.

Chemotherapy markedly reshaped the neoantigen landscape in all 8 patients (P1‐P8), hinted by small overlaps across comparison (Figure 2A, Figure S2, and Data S2). Specifically, only a small fraction of the neoantigens detected in pre‐chemotherapy tumors remained detectable post‐chemotherapy (2, 6, 4, 1, 17, 1, 11, 4 for each patient), with many new neoantigens emerging instead (8, 17, 24, 31, 11, 11, 32, 21 for each patient), while most identified neoantigens would turn invalid or faded after chemotherapy (18, 9, 25, 9, 15, 18, 26, 17 for each patient). This neoantigen dynamic was often neglected in past tumor vaccine research [47, 48, 49]. Notably, the magnitude of tumor neoantigen changes varied substantially across individuals, likely due to tumor heterogeneity and differential responses to chemotherapy.

**FIGURE 2:**
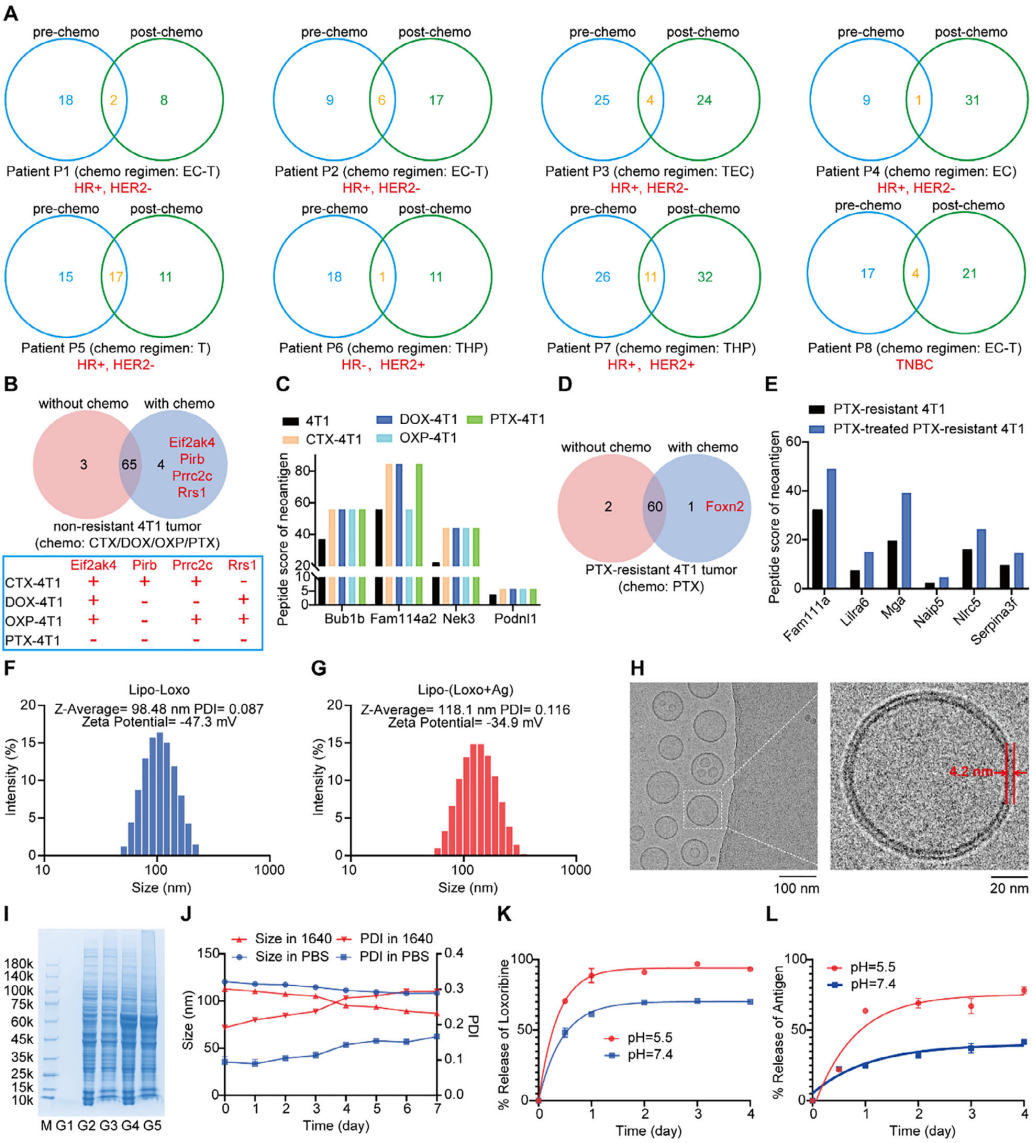
Impact of chemotherapy on TNB and characterization of the chemotherapy‐derived nanovaccine. (A) Venn diagram showing the predicted neoantigens in tumors from BC patients (P1‐P8) before and after chemotherapy (E represents epirubicin, C represents cyclophosphamide, T represents paclitaxel, H represents Herceptin, P represents pertuzumab), including 1 TNBC patient (P8). (B) Venn diagram showing the predicted neoantigens in non‐resistant murine 4T1 tumors without and with chemotherapy (cyclophosphamide (CTX), doxorubicin (DOX), oxaliplatin (OXP) or paclitaxel (PTX)), and chemotherapy‐induced emerged neoantigens were listed. Besides, the actual effects of these different chemotherapy drugs in inducing neoantigens were respectively presented. (C) Peptide score of several chemotherapy‐induced upregulated neoantigens in non‐resistant 4T1 tumors. (D) Venn diagram showing the predicted neoantigens in PTX‐resistant 4T1 tumors without and with PTX treatment, and chemotherapy‐induced emerged neoantigens were listed. (E) Peptide score of several chemotherapy‐induced upregulated neoantigens in PTX‐resistant 4T1 tumors. (F‐L) Based on tumor antigens derived from PTX‐treated PTX‐resistant 4T1 cells (PTX‐rAg), the liposomal nanovaccine Lipo‐(Loxo+PTX‐rAg) was constructed and characterized. Hydrodynamic diameter, PDI and Zeta potentials of (F) Lipo‐Loxo and (G) Lipo‐(Loxo+PTX‐rAg), measured by dynamic light scattering (DLS). (H) Cry‐TEM images of Lipo‐(Loxo+PTX‐rAg) with the thickness of lipid bilayer being labeled. (I) SDS‐PAGE analysis of the tumor antigen loading capacity of the nanovaccine (M: protein marker, G1: Lipo‐Loxo, G2: rAg, G3: Lipo‐(Loxo+rAg), G4: PTX‐rAg, G5: Lipo‐(Loxo+PTX‐rAg)). (J) Stability of Lipo‐(Loxo+PTX‐rAg) in PBS and RPMI‐1640 medium (*n* = 3). Release kinetics of (K) loxoribine and (L) antigens from Lipo‐(Loxo+PTX‐rAg) at pH 5.5 and 7.4 (*n* = 3). Data are presented as the mean ± SEM.

Meanwhile, the acquired RNA‐seq data were further analyzed to assess the impact of chemotherapy on the TME in all BC patients (P1‐P8). Kyoto Encyclopedia of Genes and Genomes (KEGG) pathway analysis of differentially expressed genes (DEGs) was first performed, confirming that chemotherapy regulated the expression levels of multiple immune‐related biological pathways in human breast tumors, including chemokine signaling, Fc gamma R‐mediated phagocytosis, and T cell receptor signaling (Figure S3). Next, immune infiltration analysis indicated that chemotherapy altered the abundance of various immune cell types within the tumors (Figures S4 and S5). Notably, across 8 patients (P1‐P8), post‐chemotherapy tumors exhibited a reduction in immunosuppressive cell subsets, including Tregs and MDSCs. However, individualized immune cell changes in response to chemotherapy seem to be a more universal phenomenon even within the same BC subtype, exemplified by different trends on intratumoral T‐cell infiltration in patients P1 and P2 compared to that of P3 and P4.

The impact of chemotherapy on immune‐related gene expression in BC patient tumors was also examined. The results suggested that the immunological effects of conventional chemotherapy might be dual‐sided due to individual differences, evidenced by upregulated multiple cell‐surface receptors (e.g., FAS, CD69, IFNGR1), suppressed immune checkpoint molecules (e.g., PDCD1, CTLA4), reduced chemokine expression (e.g., CXCL9, CXCL10), and transcription factors (e.g., STAT1, IRF1) (Figures S6 and S7).

The impact of chemotherapy on TNB was also investigated in the murine TNBC 4T1 model (Data S3). Neoantigen prediction results showed that chemotherapy partially reshaped the neoantigen landscape in non‐resistant 4T1 tumors like that in BC patients as described above (Figure 2B). In the non‐resistant 4T1 tumor model, 65 of the 68 neoantigens identified in untreated tumors remained detectable in post‐chemotherapy tumors (treated with CTX, doxorubicin (DOX), oxaliplatin (OXP) or PTX), with 4 additional neoantigens (Eif2ak4, Pirb, Prrc2c and Rrs1) emerging. Besides, the actual effect of chemotherapy in inducing new neoantigens varied across different chemotherapeutic agents. Additionally, chemotherapy also led to the upregulated peptide score of 4 neoantigens (Bub1b, Fam114a2, Nek3 and Podnl1), which indicated an upregulation of TNB (Figure 2C).

Chemotherapy induced a different spectrum of tumor neoantigens both in real‐world patient specimens and in experimental animal models. However, the clinical use of chemotherapy has often been followed by intrinsic or secondary chemo‐resistance, constituting a major challenge [50]. In our cohort, all the eight patients did not achieve pathological complete response (pCR) after chemotherapy (Figure S8). This drug‐resistant patient population typically stands for a relative worse outcome and requires ‘intensified treatment’ like CDK4/6 inhibitor, novel antibody‐drug conjugate, or other pathway inhibitors besides routinely applied systemic therapy [51, 52, 53].

We hypothesized that chemotherapy‐induced TNB upregulation might persist in chemo‐resistant tumors, which might contribute to overcoming chemoresistance and reversing innate resistance to ICIs. To test this, we established subcutaneous PTX‐resistant 4T1 tumor models and performed neoantigen prediction analysis in PTX‐resistant 4T1 tumors after different treatments. The results showed that chemotherapy still changed neoantigen landscape in PTX‐resistant 4T1 tumors. 60 of the 62 neoantigens identified in untreated PTX‐resistant tumors remained detectable in PTX‐treated PTX‐resistant tumors, with an additionally emerged neoantigen (Foxn2) (Figure 2D). PTX treatment also led to the upregulated peptide score of 6 neoantigens (Fam111a, Lilra6, Mga, Naip5, Nlrc5 and Serpina3f) in PTX‐resistant 4T1 tumors (Figure 2E).

#### 2.1.2 Preparation and Characterization of the Nanovaccine Made from Chemotherapy Upregulated Neoantigens in PTX‐Resistant Murine 4T1 TNBC Model

In our previous studies, cancer vaccines prepared from tumor antigens derived from in vitro chemotherapy‐treated cancer cells exhibited enhanced immunogenicity and initiated potent anti‐tumor immune responses [54, 55, 56]. This vaccine strategy offers potential insights into overcoming chemoresistance.

In order to maximize chemotherapy's effect on increasing TNB in chemo‐resistant tumors, PTX‐resistant 4T1 cancer cells were treated with an ultra‐high dose of chemotherapeutic agents in vitro, which would avoid the systemic high dose toxicity in vivo. Different in vitro treatments were applied on PTX‐resistant 4T1 cells to prepare tumor antigens (chemo‐rAg). Specifically, PTX‐rAg or CBP‐rAg were obtained by treating PTX‐resistant 4T1 cells with high doses of PTX or CBP, while PTX/CBP‐rAg was generated from in vitro combined treatment on PTX‐resistant 4T1 cells with PTX + CBP.

Due to the antigenic heterogeneity and cancer immunoediting, cancer vaccines based on single antigen epitope often fail to yield optimal therapeutic outcomes [57, 58, 59]. In contrast, multivalent cancer vaccines containing multiple tumor antigens can overcome these limitations by inducing a border immune response [60, 61, 62]. Additionally, antigen nanodelivery can further enhance antitumor efficacy by improving antigen presentation efficiency in antigen‐presenting cells (APCs) [63, 64, 65].

To this end, we developed a chemotherapy‐derived liposomal nanovaccine, Lipo‐(Loxo+PTX‐rAg), co‐loaded with PTX‐rAg and the acid‐responsive TLR7 agonist prodrug chol‐loxo, using the standard film‐dispersion and hydration‐sonication method [66, 67]. Following the encapsulation of PTX‐rAg, the nanovaccine showed slight increases in hydrodynamic size and zeta potential, from 98.5 nm and −47.3 mV to 118.1 nm and −34.9 mV, respectively with good uniformity (polydispersity index (PDI) ≈ 0.1) (Figure 2F,G). Cryogenic transmission electron microscopy (Cryo‐TEM) images revealed that Lipo‐(Loxo+PTX‐rAg) exhibited a homogeneous spherical morphology with an average size of approximately 100 nm (Figure 2H). The loading efficiencies of Lipo‐(Loxo+PTX‐rAg) for tumor antigens and TLR7 agonist loxoribine were calculated to be 26.63% and 15.33%, respectively, while the encapsulation efficiencies were 68.12% and 78.42%, respectively (Table S1). Sodium dodecyl sulfate‐polyacrylamide gel electrophoresis (SDS‐PAGE) analysis confirmed the successful encapsulation of tumor antigens from PTX‐resistant 4T1 cells (rAg or PTX‐rAg) into Lipo‐(Loxo+rAg) and Lipo‐(Loxo+PTX‐rAg), with no protein signals detected in the Lipo‐Loxo control group (Figure 2I). To assess stability, Lipo‐(Loxo+PTX‐rAg) was stored in phosphate buffer solution (PBS) or RPMI‐1640 medium containing fetal bovine serum (FBS) at 4°C for one week. No significant changes in particle size or PDI were observed, indicating excellent stability under physiological condition (Figure 2J). Due to the presence of the acid‐sensitive chol‐loxo prodrug in the structure, Lipo‐(Loxo+PTX‐rAg) was designed to release antigens and adjuvants in acidic environments, thereby preventing premature antigen degradation in complex physiological settings. This design enhances targeting to APCs, especially dendritic cells (DCs), which is critical for the development of effective cancer vaccines. We then investigated the release profiles of Lipo‐(Loxo+PTX‐rAg) under different conditions (Figure 2K,L). In PBS (pH = 7.4), mimicking normal physiological conditions, the release of loxoribine and antigens was relatively slow, with 70.2% of loxoribine and 41.6% of antigens released over four days. However, in acidic conditions (pH = 5.5), simulating the environment of APC endosomes [68, 69], cleavage of acid‐responsive molecules in the liposomes facilitated the release of 93.2% loxoribine and 78.2% antigens over four days, indicating enhanced antigen processing by APCs.

Together, the above results suggested that chemotherapy reshapes TNB landscape, especially PTX treatment in vitro produced 1 more neoantigen and upregulated some neoantigens’ abundance in PTX‐resistant 4T1 cell line. The derived antigens could be formulated into nanovaccine as monodispersed nanoparticles with controlled release of antigens.

### 2.2 The Nanovaccine Promotes APC Activation In Vitro and In Vivo

Effective antigen uptake and presentation by APCs are essential for initiating specific antitumor immune responses [70, 71]. As DCs represent the most critical subset of APCs, [72, 73, 74] DC2.4 cells were used to evaluate the uptake efficiency of the nanovaccine. First, Cy5.5‐labeled bovine serum albumin (Cy5.5‐BSA) was selected as a model antigen. After incubating DC2.4 cells with either Free‐(Loxo+Cy5.5‐BSA) or Lipo‐(Loxo+Cy5.5‐BSA) for 4 h, confocal laser fluorescence scanning microscopy (CLSM) was employed to assess antigen uptake (Figure 3A). The fluorescence quantitative analysis indicated that the codelivery of model antigens and adjuvants via liposomal nanovaccine significantly enhanced uptake efficiency, reaching nearly three times that of the free antigen group (Figure S9). Furthermore, the intracellular localization of the nanovaccine, Lipo‐(Loxo+Cy5.5‐rAg), was investigated after its uptake by DC2.4 cells. CLSM images revealed partial colocalization between the red fluorescence of Cy5.5‐rAg and the green fluorescence of lysosomes or endoplasmic reticulum (ER), resulting in distinct yellow fluorescence (Figure 3B). This indicates that the nanovaccine delivers tumor antigens to the ER or endosomes of APCs, where antigen epitopes are loaded onto major histocompatibility complex class I and class II (MHC‐I and MHC‐II) molecules to form MHC‐epitope complexes. These complexes are then presented to CD8^+^ and CD4^+^ T cells, inducing a potent antitumor T‐cell response. Furthermore, a methylthiazolyldiphenyl‐tetrazolium bromide (MTT) assay demonstrated no observable cytotoxicity of Lipo‐(Loxo+PTX‐rAg) in DC2.4 or RAW264.7 cells (Figure S10). Next, we assessed the in vitro activation of bone marrow‐derived dendritic cells (BMDCs) by the nanovaccine. Flow cytometry analysis of co‐stimulatory molecules CD80 and CD86, markers of DC maturation, revealed that BMDCs treated with the nanovaccine expressed significantly higher levels of these molecules compared to BMDCs treated with Free‐(Loxo+rAg). Importantly, BMDCs were more efficiently activated by nanovaccines based on chemotherapy‐induced tumor antigens (PTX‐rAg, CBP‐rAg, PTX/CBP‐rAg) than by those based on untreated tumor antigens (rAg). Specifically, co‐incubation with Lipo‐(Loxo+PTX‐rAg) induced the highest expression of CD80 and CD86, with 29% of BMDCs being double‐positive for both markers (Figure 3C and Figure S11). Additionally, the nanovaccines promoted the secretion of pro‐inflammatory cytokines by BMDCs, including tumor necrosis factor‐alpha (TNF‐α), interferon‐gamma (IFN‐γ), and interleukin‐6 (IL‐6) (Figure 3D).

**FIGURE 3:**
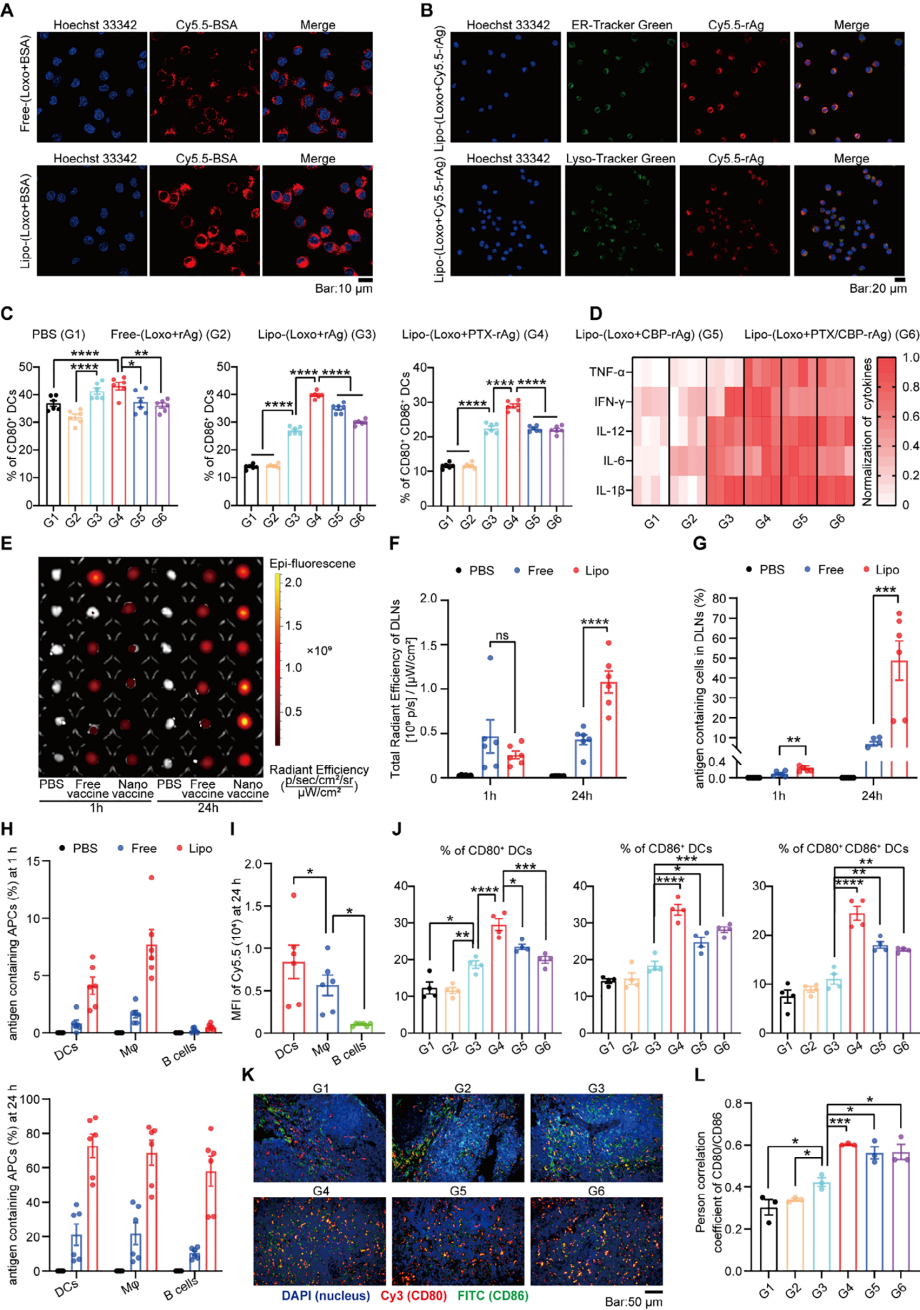
The nanovaccine promotes antigen uptake by APCs in vitro and in vivo. (A) Representative CLSM images showing the antigen uptake by DC2.4 cells after incubation with Free‐(Loxo+Cy5.5‐BSA) or Lipo‐(Loxo+Cy5.5‐BSA) for 4 h. Cy5.5‐labeled BSA (Cy5.5‐BSA, red) was used as a model antigen, nuclei were stained with Hoechst 33342 (blue) (scale bar: 10 µm). (B) Representative CLSM images showing the localization of the nanovaccine in DC2.4 cells. rAg was stained with Cy5.5 to prepare Lipo‐(Loxo+Cy5.5‐rAg) (red). After incubating DC2.4 cells with Lipo‐(Loxo+Cy5.5‐rAg) for 4 h, nuclei were stained with Hoechst 33342 (blue), while ER‐Tracker Green or Lyso‐Tracker Green was used to stain the endoplasmic reticulum (green) or endosomes (green). (scale bar: 20 µm). (C) Flow cytometry analysis of BMDC maturation in vitro after 24 h incubation with PBS, Free‐(Loxo+rAg), Lipo‐(Loxo+rAg), Lipo‐(Loxo+PTX‐rAg), Lipo‐(Loxo+CBP‐rAg) or Lipo‐(Loxo+PTX/CBP‐rAg) (*n* = 6). (D) Heatmap showing the relative cytokines secretion levels by BMDCs after different treatments. (E) Ex vivo fluorescence imaging of DLNs at 1 and 24 h after vaccination with Cy5.5‐labeled antigens (*n* = 6). (F) Quantitative analysis of fluorescence intensity (*n* = 6). (G) Flow cytometric quantification of antigen‐containing cells in DLNs at 1 and 24 h post‐injection (*n* = 6). (H) Flow cytometric quantification of antigen‐containing cells in DLNs, including DCs (CD11c^+^), macrophages (Mφ, F4/80^+^) and B cells (B220^+^), at 1 h (up) and 24 h (bottom) post‐injection (*n* = 6). (I) Cy5.5 means fluorescence intensity in DLN APC subsets at 24 h after nanovaccine administration. (J) Flow cytometric analysis of DC maturation in DLNs 24 h after subcutaneous injection of PBS, Free‐(Loxo+rAg), Lipo‐(Loxo+rAg), Lipo‐(Loxo+PTX‐rAg), Lipo‐(Loxo+CBP‐rAg) or Lipo‐(Loxo+PTX/CBP‐rAg) (*n* = 6). (K) Representative immunofluorescence images of DLNs. Nuclei were stained with DAPI (blue), CD80 (Cy3, red) and CD86 (FITC, green) are shown, with colocalization indicated in orange. (L) Colocalization coefficient of the costimulatory molecules CD80 and CD86 in DLNs (*n* = 3). Data are presented as the mean ± SEM. Statistical significance was calculated by unpaired t‐test (^*^ *p* < 0.05, ^**^ *p* < 0.01, ^***^ *p* < 0.001, ^****^ *p* < 0.0001).

The in vivo effect of nanovaccine on enhancing antigen uptake was further investigated in lymph nodes, the primary location of antigen presentation [75]. Mice were subcutaneously vaccinated with either the nanovaccine Lipo‐(Loxo+Cy5.5‐BSA) or a free mixture of adjuvants and antigens Free‐(Loxo+Cy5.5‐BSA). At different time points post‐administration, draining lymph nodes (DLNs) near the injection site were isolated, and analyzed for fluorescence intensity with an in vivo imaging system (IVIS) (Figure 3E). Lipo‐(Loxo+Cy5.5‐BSA) entered DLNs as early as 1 h post administration and accumulated significantly by 24 h (Figure 3F). In contrast, the free vaccine group exhibited slightly higher fluorescence at 1 h, likely due to the smaller size of free Cy5.5‐BSA facilitating particle diffusing. However, free BSA was cleared more quickly from DLNs, as evidenced by a much stronger fluorescence signal in the nanovaccine group at 24 h.

Flow cytometry was then used to quantify antigen uptake in the DLNs. As shown in Figure 3G, the proportion of Cy5.5^+^ cells in the DLNs was significantly higher in the Lipo‐(Loxo+Cy5.5‐BSA) group compared to the Free‐(Loxo+Cy5.5‐BSA) group, demonstrating that nano‐delivery improves antigen uptake by resident APCs in the DLNs. Given the role of APCs in initiating antitumor T‐cell immunity, antigen uptake by specific APC subsets was further analyzed. All three major APC subsets in the DLNs (DCs, macrophages, and B cells) showed significantly higher antigen uptake in the nanovaccine group compared to the free vaccine group (Figure 3H and Figure S12). Importantly, about 70% of DCs in the Lipo‐(Loxo+Cy5.5‐BSA) group phagocytosed the nanovaccine, nearly three times higher than in the Free‐(Loxo+Cy5.5‐BSA) group. In the Lipo‐(Loxo+Cy5.5‐BSA) group, the average Cy5.5 fluorescence intensity in macrophages and B cells was significantly lower than that in DCs, confirming roles of DCs as the most potent APCs (Figure 3I).

In DLNs, mature DCs present antigens, express co‐stimulatory molecules, and secrete specific cytokines to activate T cells, making them pivotal mediators of antitumor immune responses [74]. To investigate the in vivo DC activation potential of the nanovaccines, mice were subcutaneously injected with nanovaccines based on different chemo‐rAgs. Flow cytometric analysis of the DLNs showed a trend consistent with in vitro findings (Figure 3J and Figure S13). Specifically, Lipo‐(Loxo+rAg) promoted DC maturation more effectively than Free‐(Loxo+rAg). Furthermore, due to their higher immunogenicity, nanovaccines based on chemo‐rAg (PTX‐rAg, CBP‐rAg and PTX/CBP‐rAg) induced significantly enhanced DC maturation, consistent with immunofluorescence (IF) staining of mouse DLNs (Figure 3K). Among the different chemo‐rAg‐based nanovaccines, Lipo‐(Loxo+PTX‐rAg) induced the highest DC maturation rate in DLNs, as evidenced by the highest levels of CD80 and CD86 expression (Figure 3L).

### 2.3 In Vivo Biosafety of The Nanovaccine

Conventional chemotherapeutics often exhibit side effects and cause damage to normal tissues due to their lack of tumor‐specificity [76, 77]. For example, PTX inhibits cancer cell proliferation by disrupting tubulin depolymerization, but it is associated with significant clinical side effects, including hematopoietic suppression and hepatotoxicity. To evaluate the biosafety of the nanovaccine, healthy mice were subcutaneously injected with PBS or Lipo‐(Loxo+PTX‐rAg) on days 0, 3 and 6. In the positive control group, mice were injected with PTX via the tail vein following the same schedule. Whole blood samples were collected on days 1 and 7, and blood parameters including white blood cell (WBC) count, red blood cell (RBC) count, hematocrit (HCT), hemoglobin (HGB), mean corpuscular volume (MCV), and platelets (PLT) count were analyzed. As shown in Figure 4A,B, PTX treatment resulted in a significant reduction in WBC and RBC counts, indicating marked hematopoietic suppression. In contrast, no significant changes in blood cell parameters were observed in mice receiving one or three doses of Lipo‐(Loxo+PTX‐rAg) compared to the PBS group. One week after the third treatment, serum biochemical markers, including aspartate aminotransaminase (AST), alanine aminotransferase (ALT), blood urea nitrogen (BUN), and creatinine (CRE), were examined to assess hepatotoxicity and nephrotoxicity (Figure 4C). Histological analysis was also performed on heart, liver, spleen, lung and kidney tissues using hematoxylin and eosin (H&E) staining (Figure 4D). Mice treated with PTX showed significantly elevated serum ALT levels, and histological examination revealed hepatocellular swelling and marked morphological abnormalities, indicating substantial hepatotoxicity. In contrast, mice treated with Lipo‐(Loxo+PTX‐rAg) exhibited no abnormal serum or tissue level toxicity, demonstrating that the nanovaccine based on chemo‐rAg has favorable biosafety compared to traditional chemotherapy.

**FIGURE 4:**
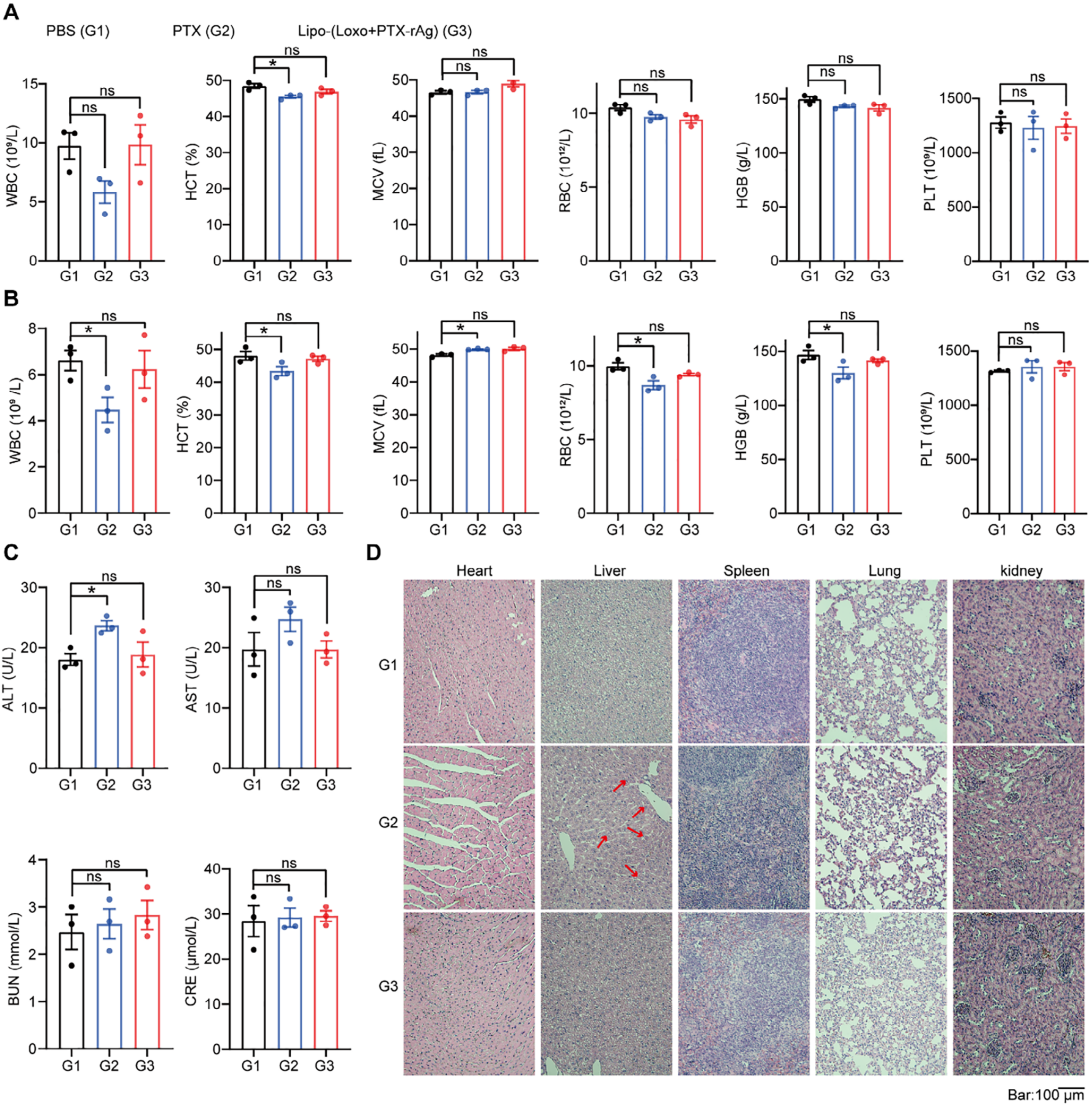
Biosafety assessment of the nanovaccine. Hematological parameters, including HCT, MCV, RBC, HGB, WBC, were measured in mice after (A) a single administration or (B) multiple administrations of PBS (i.v.), PTX (i.v.) or Lipo‐(Loxo+PTX‐rAg) (s.c.) (*n* = 3). (C) Serum biochemistry markers of hepatic and renal function, including ALT, AST, BUN and CRE, were assessed one week after the final vaccination (*n* = 3). (D) Representative H&E staining of major organs (heart, liver, spleen, lung and kidney) following different treatments. Data are presented as the mean ± SEM. Statistical significance was calculated by unpaired t‐test (^*^ *p* < 0.05, ^**^ *p* < 0.01, ^***^ *p* < 0.001, ^****^ *p* < 0.0001).

### 2.4 The Nanovaccine Overcomes PTX Resistance and the Resistance of anti‐PD‐L1 in PTX‐Resistant Murine 4T1 TNBC Model

To evaluate the therapeutic efficacy of the nanovaccine, a subcutaneous non‐resistant TNBC 4T1 tumor model was first established in female BALB/c mice. Most previously studies on therapeutic cancer vaccines administer the first vaccination as early as day 3 after tumor implantation, when tumors are typically non‐palpable [45, 78, 79, 80]. In this study, vaccination was initiated when tumors reached a visible size (∼ 50–100 mm^3^), representing a more clinically relevant challenge, which is also much more difficult to treat. There are few cancer vaccine studies starting for this tumor volume. Lipo‐(Loxo+PTX‐Ag), which was formulated with tumor antigens derived from PTX‐treated non‐resistant 4T1 cells (PTX‐Ag), more effectively delayed tumor progression than the corresponding free formulation, Free‐(Loxo+PTX‐Ag) (Figure 5A). No significant body‐weight loss was observed (Figure S14), confirming the favorable biosafety profile of the nanovaccine.

**FIGURE 5:**
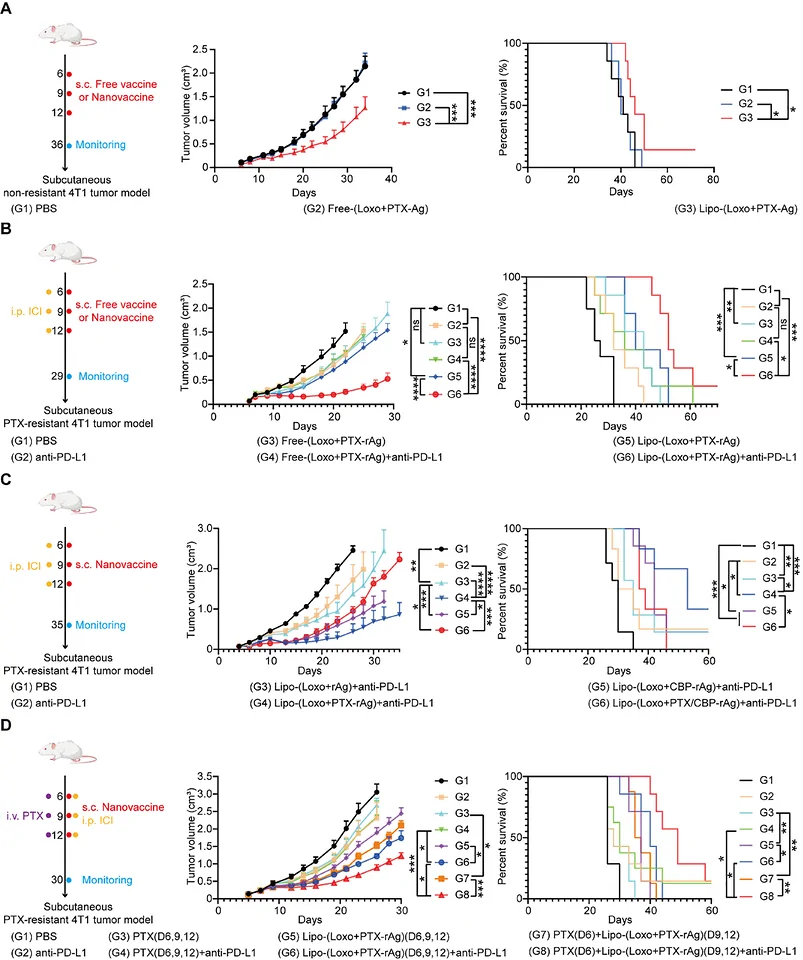
The nanovaccine reverses the innate resistance of ICI and overcomes PTX resistance in the PTX‐resistant tumor model. (A‐D) Schematic illustration of the treatment schedule, along with the corresponding average tumor growth and survival curves in mice bearing non‐resistant or PTX‐resistant 4T1 tumors. (A) Investigation into whether the nanovaccine could inhibit tumor growth in the non‐resistant 4T1 model (*n* = 7). (B) Verification if the nanovaccine could reverse the innate resistance of anti‐PD‐L1 (*n* = 7–8). (C) Studies regarding different chemo‐rAg based nanovaccines in reversing the innate resistance of anti‐PD‐L1 (*n* = 6–7). (D) Verification if our designed nanovaccine could overcome drug resistance (*n* = 7–8). Data are presented as the mean ± SEM. Tumor growth curves were analyzed by two‐way ANOVA with Geisser‐Greenhouse correction. Statistical differences of survival time were calculated by Kaplan Meier method, and the p value was calculated by Gehan‐Breslow‐Wilcoxon test (^*^ *p* < 0.05, ^**^ *p* < 0.01, ^***^ *p* < 0.001, ^****^ *p* < 0.0001).

Later, the antitumor efficacy of this vaccine strategy was further verified if it could reverse the innate resistance of ICI in subcutaneous PTX‐resistant 4T1 tumor model with a starting tumor size around 50–100 mm^3^. Tumor growth curves showed that Lipo‐(Loxo+PTX‐rAg) significantly delayed PTX‐resistant 4T1 tumor progression compared to PBS, whereas a simple mixture of free antigens and adjuvants Free‐(Loxo+PTX‐Ag) failed to exert similar effects (Figure 5B). Furthermore, the nanovaccine could reverse the innate resistance of anti‐PD‐L1, thus the combination of the nanovaccine and anti‐PD‐L1 enhanced tumor growth inhibition and prolonged survival compared with either Lipo‐(Loxo+PTX‐rAg) or anti‐PD‐L1 monotherapy. Notably, one mouse in the combination treatment group achieved complete tumor regression (Figure S15).

Subsequently, we also investigated the efficacy of different chemo‐rAg based nanovaccines (Figure 5C and Figure S16). Tumor antigens were derived from untreated PTX‐resistant 4T1 cells (rAg), PTX‐ or CBP‐treated PTX‐resistant 4T1 cells (PTX‐rAg and CBP‐rAg), and PTX+CBP co‐treated PTX‐resistant 4T1 cells (PTX/CBP‐rAg). Chemo‐rAg‐based nanovaccines outperformed rAg‐based formulation in inhibiting PTX‐resistant tumor growth and prolonging survival. Among these different chemo‐rAg, the PTX‐rAg‐based nanovaccine demonstrated superior antitumor activity, consistent with its superior ability to promote DC maturation in vitro and in vivo.

Furthermore, to verify if our designed nanovaccine could overcome drug resistance, we first compared the efficacy between nanovaccine and PTX treatment. Although PTX markedly inhibited growth of non‐resistant 4T1 tumors (Figure S17), it showed minimal therapeutic benefit in PTX‐resistant 4T1 tumors, either alone or combined with anti‐PD‐L1. However, our results showed that nanovaccine only group (G5) could overcome the PTX resistance and showed significant prolonged survival than that of PTX only (G3) (Figure 5D and Figure S18). Combination of the nanovaccine plus anti‐PD‐L1 (G6) also displayed better efficacy than that of PTX plus anti‐PD‐L1 (G4).

To verify if this efficacy of nanovaccine was produced from unleashed antigens, we replaced the initial nanovaccine administration with PTX to induce in situ tumor antigens release and investigated the impact on antitumor efficacy. Importantly, replacing the first vaccination with PTX+ Lipo‐(Loxo+PTX‐rAg) (G7) slightly improved the efficacy of Lipo‐(Loxo+PTX‐rAg) (G5), while it remained the significant enhanced efficacy and prolonged survival in comparison to PTX only group (G3). This enhancement was significantly potentiated by anti‐PD‐L1 combination (G8). A plausible explanation is that PTX promotes in situ release of tumor antigens, particularly neoantigens, thereby helping to relieve the immunosuppressive TME and elicit more robust antitumor immune responses, thus reversing the innate resistance of anti‐PD‐L1 in murine TNBC.

In summary, these results suggested that the nanovaccine could reverse the innate resistance of anti‐PD‐L1 and overcome the PTX resistance on murine resistant TNBC tumor model.

### 2.5 Antitumor Efficacy of Chemotherapy‐Derived Nanovaccine is Related with Upregulated Neoantigens

In order to investigate the antitumor mechanism of chemotherapy‐derived nanovaccine, proteomics analysis was performed on multiple tumor antigen samples. First, the results revealed significant changes in the protein expression profile of 4T1 cells following the development of PTX resistance (Figure 6A). Compared to antigens derived from untreated PTX‐resistant 4T1 cells (rAg), chemo‐rAg (PTX‐rAg, CBP‐rAg and PTX/CBP‐rAg) displayed substantial differences in protein composition (Figure 6B), which might be caused by the changes of multiple upstream stress pathways, including ER stress, the unfolded protein response, and autophagy (Figure S19). Notably, the expression levels of several DAMPs (e.g., Hsp90ab1, Hmgb1, Capza2, Carmil1, Hspa13, Bcl2l13, Macroh2a1) (Figure 6C and Table S2), multiple typical cancer‐related proteins (e.g., Cenpf, Muc5Ac, Polr2a), metastasis‐associated proteins (e.g., Msln, Sema3e, Ect2, Srsf3, Foxm1, Plk1) and a testis cancer antigen (Pbk) were significantly upregulated in chemo‐rAg (Figure 6D) [81, 82]. Additionally, mining our proteomic analysis data for previously reported neoantigen sequences (Data S4), [82, 83, 84] it revealed that 4 neoantigen peptides (Dync1h1, Actn2, Qars, and Ilkap) were significantly more abundant in chemo‐rAg in comparison to rAg (Figure 6E and Table S3). Surprisingly, these 4 neoantigens were also detected in murine 4T1 tumors treated with different chemotherapeutics (Data S5).

**FIGURE 6:**
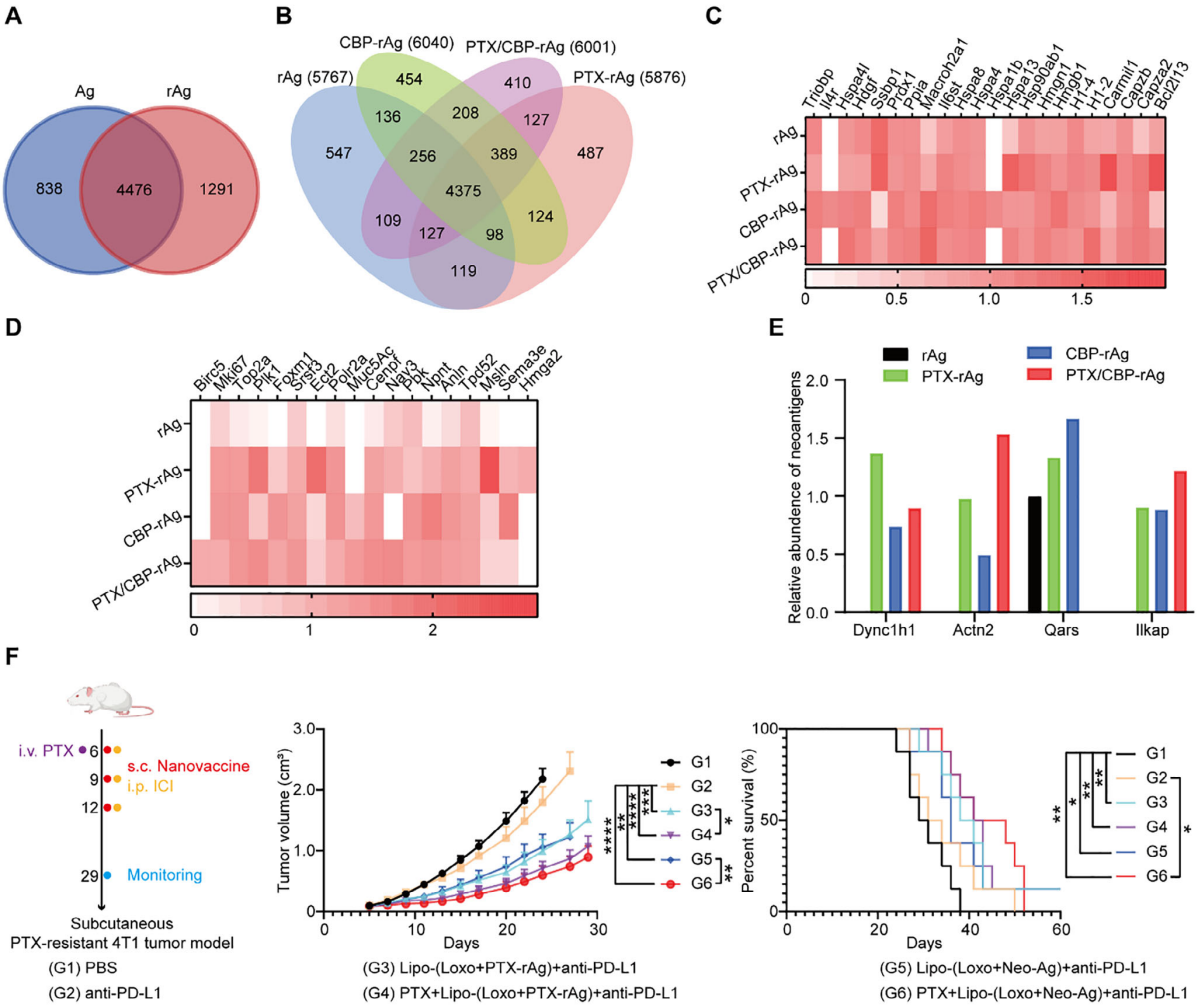
The mechanism of chemotherapy‐derived nanovaccines. (A) Venn diagram showing the tumor antigens derived from non‐resistant 4T1 cells (Ag) and PTX‐resistant 4T1 cells (rAg). (B) Venn diagram showing the tumor antigens derived from chemotherapy‐treated PTX‐resistant 4T1 cells (including PTX‐rAg, CBP‐rAg and PTX/CBP‐rAg), with rAg as the control. (C) Heatmap showing the relative abundance of DAMPs in rAg, PTX‐rAg, CBP‐rAg and PTX/CBP‐rAg. (D) Heatmap showing the relative abundance of tumor‐associated antigens (TAAs) in rAg, PTX‐rAg, CBP‐rAg and PTX/CBP‐rAg. (E) Relative abundance of several neoantigens in rAg, PTX‐rAg, CBP‐rAg and PTX/CBP‐rAg. (F) Based on two PTX‐upregulated neoantigen peptides (Actn2 and Dync1h1), the neoantigen nanovaccine Lipo‐(Loxo+Neo‐Ag) was prepared. And the antitumor efficacy of Lipo‐(Loxo+PTX‐rAg) and Lipo‐(Loxo+Neo‐Ag) was compared in the PTX‐resistant 4T1 model, with or without combined anti‐PD‐L1 and PTX treatment (*n* = 8). Data are presented as the mean ± SEM. Tumor growth curves were analyzed by two‐way ANOVA with Geisser‐Greenhouse correction. Statistical differences of survival time were calculated by Kaplan Meier method and the p value was calculated by Gehan‐Breslow‐Wilcoxon test (^*^ *p* < 0.05, ^**^ *p* < 0.01, ^***^ *p* < 0.001, ^****^ *p* < 0.0001).

Based on the comprehensive analysis of the above results, there seems to be a positive association between the therapeutic efficacy of tumor antigen‐based nanovaccines and neoantigen abundance. For example, Lipo‐(Loxo+PTX‐rAg) exhibited the strongest antitumor effects while PTX‐rAg contained the highest neoantigen load. This observation is consistent with numerous studies indicating that a higher neoantigen load is often associated with improved clinical outcomes in cancer patients [85, 86, 87]. Given the crucial role of neoantigens in eliciting tumor‐specific immune responses, we hypothesized that the superior efficacy of chemo‐rAg‐based nanovaccines is primarily driven by increased neoantigen expression.

To test this hypothesis, we prepared a neoantigen nanovaccine Lipo‐(Loxo+Neo‐Ag) using two PTX‐upregulated neoantigens (Actn2 and Dync1h1) and evaluated the in vivo antitumor efficacy (Figure 6F and Figure S20). The neoantigens‐based nanovaccine exhibited comparable inhibitory effects on the growth of PTX‐resistant 4T1 tumor to the PTX‐rAg‐based formulation. The addition of PTX to induce in situ tumor antigen release further enhanced the antitumor effects of both nanovaccines. Notably, when combined with anti‐PD‐L1 and PTX, both Lipo‐(Loxo+Neo‐Ag) and (Loxo+PTX‐rAg) significantly delayed tumor progression and improved survival compared to anti‐PD‐L1 monotherapy. Besides, based on the other 4 neoantigens (Foxn2 Fam111a, Mga, and Nlrc5) which were obtained through corresponding prediction algorithm (Figure 2D,E), we prepared another nanovaccine Neo‐VAX. It also showed an excellent antitumor efficacy in the PTX‐resistant 4T1 tumor model (Figure S21).

Therefore, these results reinforce the nanovaccine from PTX resistance could be utilized to overcome the innate resistance of anti‐PD‐L1 and PTX resistance.

### 2.6 The Nanovaccine Triggers Specific T‐Cell Responses and Reverses the Immunosuppressive TME when Combined with Anti‐PD‐L1

Antigen‐specific CD4^+^ and CD8^+^ T cells play a critical role in tumor immunity by eliminating cancer cells and inhibiting tumor growth [88, 89]. We first investigated ex vivo T cell proliferation via carboxyfluorescein diacetate succinimidyl ester (CFSE) dilution assay following co‐incubation of BMDCs with different nanovaccines. Flow cytometric analysis revealed that the nanovaccine significantly promoted T cell proliferation compared with Free‐(Loxo+rAg) (Figure 7A–C and Figure S22). To assess the capacity of nanovaccines formulated with different tumor antigens to induce antigen‐specific T cell responses, we analyzed ex vivo IFN‐γ secretion from splenocytes. One week after the final treatment, splenocytes were harvested from mice and incubated with corresponding tumor antigens. IFN‐γ production was then measured using flow cytometric analysis (Figure S23). Analysis of IFN‐γ secretion by CD4^+^ or CD8^+^ T cells showed that CD4^+^ T cell activation was largely comparable among treatment groups (Figure 7D). Furthermore, combination treatment with anti‐PD‐L1 and Lipo‐(Loxo+rAg) or Lipo‐(Loxo+PTX‐rAg) enhanced CD8^+^ T cell activation relative to PBS or anti‐PD‐L1 monotherapy. Surprisingly, Lipo‐(Loxo+Neo‐Ag) combined with anti‐PD‐L1 showed promising effects only when further combined with PTX (Figure 7E).

**FIGURE 7:**
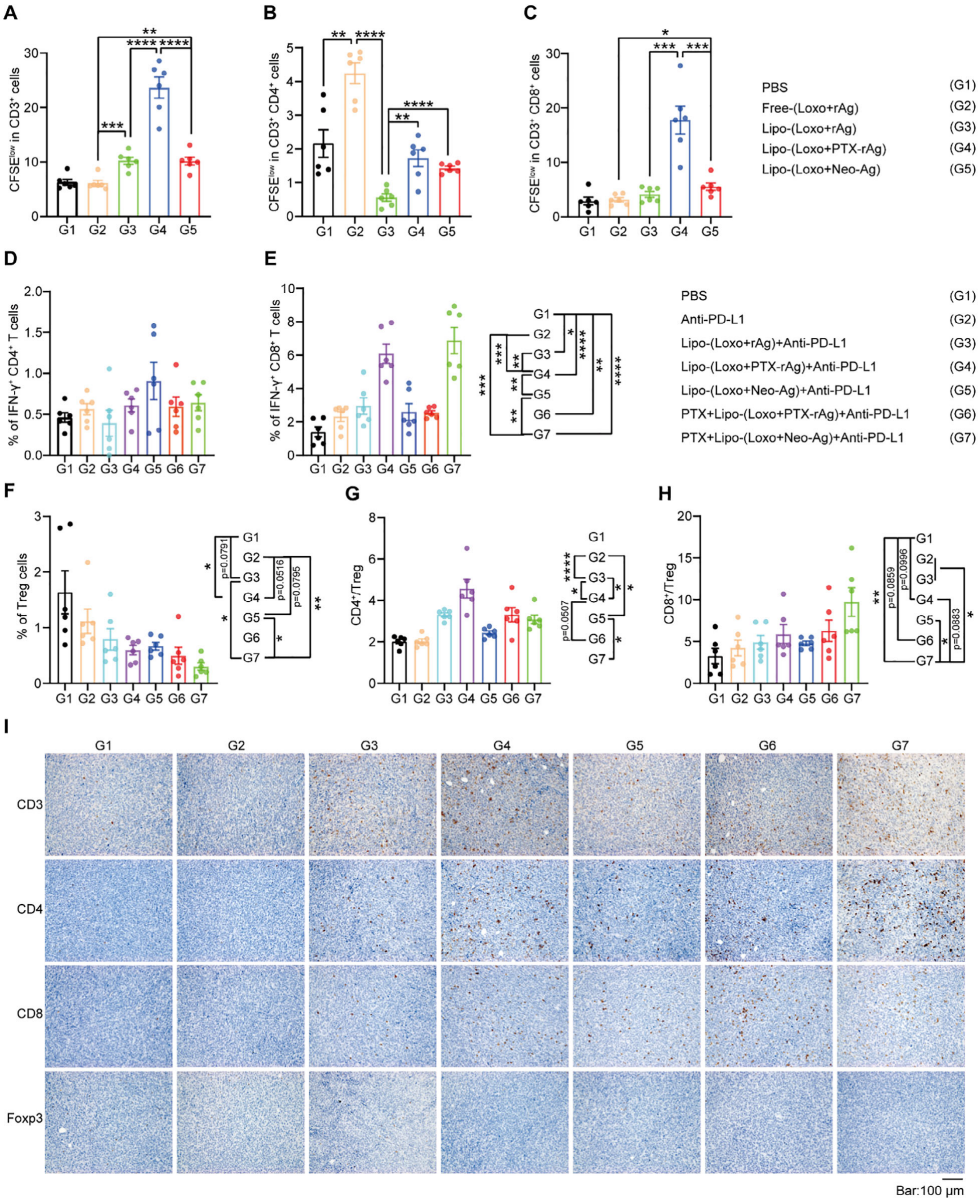
Combination therapy with the nanovaccine and anti‐PD‐L1 alleviates immunosuppressive TME. (A‐C) Flow cytometry analysis of splenocyte proliferation, including (A) CD3^+^, (B) CD3^+^ CD4^+^ and (C) CD3^+^ CD8^+^ cells, after co‐incubation with BMDCs and different antigen formulations (*n* = 6). (D‐E) Flow cytometric analysis of (D) IFN‐γ^+^ CD8^+^ T cells and (E) IFN‐γ^+^ CD4^+^ T cells in splenocytes pulsed ex vivo with the corresponding tumor antigens (*n* = 6). (F‐H) Flow cytometric analysis of intra‐tumoral immune populations, showing the relative abundance of (F) Treg cells (Foxp3^+^ CD4^+^), (G) the CD4^+^ T/Treg ratio, and (H) the CD8^+^ T/Tregs ratio (*n* = 6). (I) Representative immunohistochemical images of tumor sections showing infiltration of CD3^+^, CD4^+^, CD8^+^ and Foxp3^+^ cells after different treatments (*n* = 3). Tumors were collected one week after the final treatment. Data are presented as the mean ± SEM. Statistical significance was calculated by unpaired t‐test (^*^ *p* < 0.05, ^**^ *p* < 0.01, ^***^ *p* < 0.001, ^***^ *p* < 0.0001).

Intratumoral T cell infiltration is a crucial prerequisite for potent antitumor immunity [90]. Therefore, we investigated the impact of nanovaccines on intratumoral T cell infiltration. Flow cytometry analysis showed that the combination treatment with nanovaccines and anti‐PD‐L1 significantly reduced Treg abundance (Figure 7F), and increased the CD4^+^ T/Treg ratio (Figure 7G) and CD8^+^ T/Treg ratio (Figure 7H). Notably, Lipo‐(Loxo+PTX‐rAg) and Lipo‐(Loxo+Neo‐Ag) exhibited more potent effects than Lipo‐(Loxo+rAg), likely due to their higher immunogenicity. The addition of PTX further enhanced these effects. Immunohistochemical (IHC) analysis of tumor sections showed that nanovaccines effectively increased the intratumoral CD4^+^ and CD8^+^ T cell infiltration while reducing Treg proportions (Figure 7I), contributing to the alleviation of the immunosuppressive TME [91, 92].

We further performed RNA‐seq on mouse tumor tissues to characterize the effects of different treatments on the TME (Figure S24). Using gene sets from the Molecular Signatures Database (MSigDB), gene set enrichment analysis (GSEA) revealed that combination therapy upregulated several pro‐immune pathways (e.g., IFN‐γ response, inflammatory response, and TNF‐α signaling) and downregulated the immunosuppressive TGF‐β signaling pathway (Figure S24A). Additionally, we observed significantly increased expression of immune‐related genes in the combination treatment group (Figure S24B), including chemokines (Ccl3, Ccl4, and Cxcl9), chemokine receptors (Ccr7 and Cxcr3), cytotoxic effector molecules (Fas, Stat1, Tnfsf10), and activation markers (Il2rb, Klrc2, Klrd1, Nkg7, Tbx21). To further assess the TME components, we applied the Estimation of Stromal and Immune Cells in Malignant Tumor Tissues Using Expression Data (ESTIMATE) algorithm [93]. The nanovaccine plus anti‐PD‐L1 group showed lower tumor purity (Figure S24C) and higher immune scores (Figure S24D), indicating enhanced immune cell infiltration into the TME. This group also exhibited significantly higher ESTIMATE scores (Figure S24E). Using the Immune Cell Abundance Identifier for Mouse (ImmuCellAI‐mouse) algorithm, [94] we confirmed that combination treatment induced improved immune cell infiltration, with greater abundance of DCs and T cells, and reduced abundance of Treg cells (Figure S24F–Z).

### 2.7 The Nanovaccine Induces Long‐Lasting Immunological Memory

To evaluate the durability of immune response induced by the liposome nanovaccine, we first performed a tumor rechallenge experiment. Cured mice that had experienced sustained tumor regression for 90 days following combination treatment with the nanovaccine and anti‐PD‐L1 were rechallenged with PTX‐resistant 4T1 tumors in the contralateral flank (Figure 8A). All cured mice successfully rejected the rechallenged tumor, whereas naïve mice exhibited rapid tumor growth, with 100% mortality within 30 days (Figure 8B,C and Figure S25).

**FIGURE 8:**
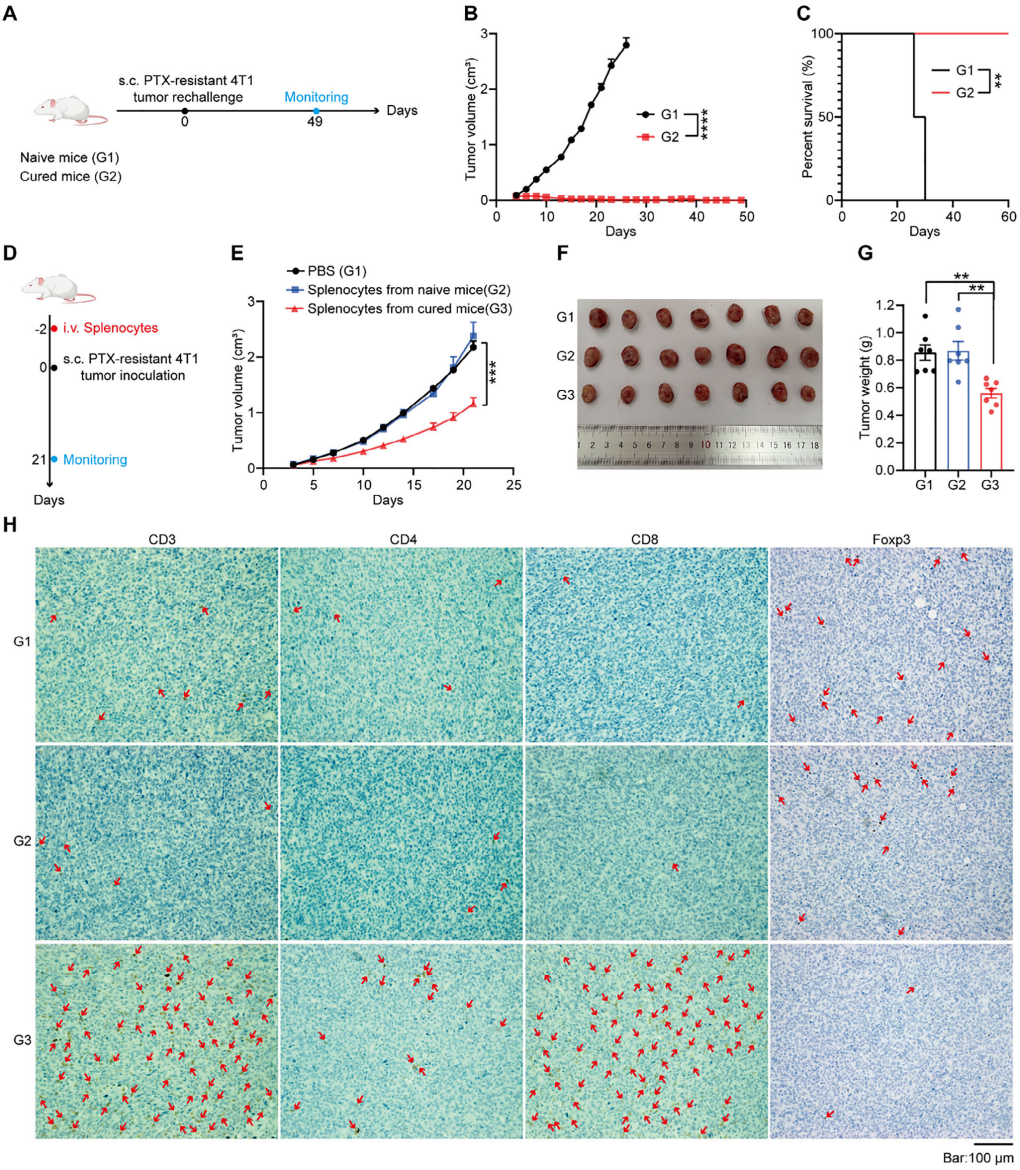
The nanovaccine induces durable immunological memory. (A) Schematic illustration of the tumor rechallenge experiment. (B) Tumor growth curves and (C) survival curves of cured mice versus naïve mice after tumor inoculation (*n* = 4–5). (D) Schematic illustration of the adoptive cell transfer experiment. Mice were euthanized on day 21, and tumors were collected for subsequent analysis (*n* = 7–8). (E) Tumor growth curves of recipient tumor‐bearing mice treated with PBS, splenocytes from cured mice, or splenocytes from naïve mice. (F) The tumor pictures and (G) corresponding quantification in different treatment groups. (H) Representative immunohistochemical images of tumor sections showing infiltration of CD3^+^, CD4^+^, CD8^+^ and Foxp3^+^ (Treg) cells after different treatments (*n* = 3). Positive cells are marked by red arrows. Data are presented as the mean ± SEM. Tumor growth curves were analyzed by two‐way ANOVA with Geisser‐Greenhouse correction. Statistical differences of survival time were calculated by Kaplan Meier method and the *p* value was calculated by Gehan‐Breslow‐Wilcoxon test (^*^ *p* < 0.05, ^**^ *p* < 0.01, ^***^ *p* < 0.001, ^****^ *p* < 0.0001).

Next, we conducted an adoptive splenocyte transfer experiment. Splenocytes were harvested from naïve or cured donor mice, and 4 × 10^6^ splenocytes were intravenously injected into non‐immunized recipient mice. Two days later, 5 × 10^5^ PTX‐resistant 4T1 cells were subcutaneously implanted into the right flank of recipient mice (Figure 8D). Transfer of splenocytes from cured donors markedly suppressed tumor growth compared with PBS treatment or transfer of splenocytes from naïve donors (Figure 8E–G and Figure S26). Additionally, IHC revealed significantly increased intratumoral infiltration of CD4^+^ and CD8^+^ T cells, and a concomitant reduction in Foxp3^+^ T cells in i recipients of cured splenocytes compared to naïve controls (Figure 8H). Collectively, these results demonstrate that combined treatment with the liposomal nanovaccine and anti‐PD‐L1 induces durable and potent immunological memory.

## 3 Conclusions

Chemotherapy is often limited by severe off‐target toxicity and the development of chemoresistance [6, 7, 8, 12]. For example, as a frontline treatment for TNBC, chemotherapy elicits sustained responses in only a small subset of patients, while most eventually develop drug resistance, leading to rapid tumor progression and poor prognosis [9, 10, 11, 13, 14]. The mechanisms underlying tumor drug resistance are complex and incompletely understood, with proposed explanations including increased drug excretion, metabolism reprogramming, enhanced DNA damage repair (particularly relevant to platinum‐based chemotherapeutics that target DNA), and genetic/epigenetic alterations (e.g., mutations in genes encoding drug target proteins) [15, 95, 96, 97]. Based on these mechanisms, neoadjuvant therapy including related chemotherapeutics, like platinum, is considered standard of care for TNBC [3, 8]. Although platinum monotherapy yields a higher objective response rate (ORR) in mTNBC patients with BRCA1/2 mutation than in those with wild type BRCA1/2, [95] integrating platinum drugs into neoadjuvant regimens only increases pCR rates by around 15%, [96] which remains suboptimal. Another straightforward strategy involves maintaining sufficiently high intratumoral drug concentration by enhancing drug uptake or inhibiting efflux, though this often causes severe side effects due to poor specificity [97]. Multi‐drug chemotherapy is commonly used in clinic practice, [98] but in the worst‐case scenario, the emergence of multidrug resistance can render such combinations ineffective [99]. Antibody‐drug conjugates (ADCs), which rely on specific tumor targeting, have shown great potential in TNBC treatment [100, 101]. However, strategies targeting known chemoresistance mechanisms often exhibit limited or unsatisfactory efficiency, highlighting the persistent challenge of overcoming drug resistance and the need for effective resistance‐reversing solutions.

Chemotherapy also modulates host immune responses in complex ways. High‐dose of CTX suppresses immune responses in mTNBC, and neoadjuvant chemotherapy with nab‐PTX followed by epirubicin and CTX depletes most B cells while reducing the frequency of natural killer (NK) cells and CD4^+^ T lymphocytes in TNBC patients [102]. Although the relative abundance of TILs is a prognostic factor for improved survival in early‐stage TNBC, [103] chemotherapy often reduces or depletes lymphocytes in BC patients [104, 105]. T cell exhaustion has also been observed in other cancer types [33, 34]. Beyond these immunosuppressive effects, chemotherapy exerts immunostimulatory effects as well, such as depleting immunosuppressive cells (e.g., Tregs and MDSCs), [106] activating DCs and increasing infiltration of CTLs [39]. Critically, chemotherapy can induce ICD and reshape the immunosuppressive TME, thereby enhancing tumor antigenicity and adjuvanticity [42, 107, 108].

For tumors with acquired chemoresistance, continued high‐dose drug administration becomes ineffective, leading to severe toxicity, and exacerbates immunosuppression. Notably, chemotherapy can increase TMB, a phenomenon that remains incompletely characterized [43]. Mutational signatures serve as markers of cancer cell drug sensitivity [109]. TNB, which reflects TMB and generates immunogenic neoepitopes, is elevated in some tumor types following chemoradiotherapy [110, 111, 112, 113]. Given that TMB is a predictive biomarker for response to ICIs, [18, 114] we hypothesized that chemotherapy may upregulate TMB and TNB in PTX‐resistant TNBC, thereby increasing neoantigen production and enhancing cancer immunotherapy.

In this study, the analysis of neoantigen prediction performed on tumor samples from clinical BC patients indicated that chemotherapy significantly altered the neoantigen landscape in the tumors. Similar findings were observed in both murine non‐resistant and resistant 4T1 tumor models. Additionally, chemotherapy displayed the ability to increase TNB, which might contribute to overcoming drug resistance. Further proteomic analysis revealed that in vitro chemotherapy‐induced tumor antigens (chemo‐rAg) exhibited superior immunogenicity, as evidenced by higher levels of DAMPs and neoantigens. Then, we developed therapeutic nanovaccines using those chemo‐rAg. Subsequent experimental results showed that the nanovaccines, especially PTX‐rAg‐based Lipo‐(Loxo+PTX‐rAg), effectively promote APC activation and subsequent antigen‐specific T cell responses, ultimately enhancing antitumor immunity and overcoming drug resistance. Incorporating PTX chemotherapy and ICIs into the treatment regimen further improved antitumor efficacy. Mechanically, the nanovaccine enhanced intratumoral T cell infiltration and alleviated the immunosuppressive TME in 4T1 tumors. Interestingly, replacing chemo‐rAg with two chemotherapy‐upregulated neoantigens (Actn2 and Dync1h1) resulted in a nanovaccine with similar antitumor efficacy.

Therapeutic cancer vaccines have garnered considerable attention for their ability to activate the immune system and induce a potent and long‐lasting antitumor response [115, 116, 117, 118, 119, 120]. The efficacy of cancer vaccine depends largely on the selection of tumor antigens [16]. Current vaccines primarily target TAAs and tumor‐specific antigens (TSAs). However, their efficacy requires further investigation, as overall responses remain suboptimal [16, 17, 18, 19]. This is partly due to insufficient antigen selection, which fails to elicit effective antigen‐specific T cell responses. Moreover, tumors undergo immunoediting, leading to the loss of highly immunogenic antigens, rendering corresponding vaccines ineffective [20]. Neoantigens, which are TSAs derived from nonsynonymous mutations and other genetic alterations, exhibit higher immunogenicity, specificity and MHC affinity than TAAs, making them promising targets for therapeutic vaccines [21, 22]. Despite progresses in several tumor types, neoantigen‐based cancer vaccines have not demonstrated definitive efficacy in most clinical trials [23, 24, 25]. Additionally, neoantigen screening is complex, time‐consuming, and costly, introducing variability into neoantigen prediction [20, 26, 27]. Progress has also been made in BC vaccine development [121, 122, 123, 124, 125, 126]. For example, the HER2/neu‐derived peptide vaccine GP2 has shown excellent clinical efficacy in BC patients [127, 128]. Combinational therapy with GP2 and GM‐CSF elicits potent immune responses and achieves 100% disease‐free survival with minimal toxicity in HER2/neu‐positive patients receiving adjuvant trastuzumab [129, 130]. However, most current BC vaccines target HER2 or HER2‐derived peptide, whose low expression in TNBC limits their utility in this subtype [131, 132, 133, 134].

In conclusion, unlike traditional strategies for overcoming drug resistance, such as enhancing drug uptake, inhibiting drug efflux, combination therapies, and ADCs, our study successfully leveraged PTX‐resistant 4T1 cells to develop nanovaccines from an immunological perspective. This approach not only enhances cancer immunotherapy efficacy but also expands the potential of therapeutic cancer vaccines as a promising strategy to overcome drug resistance. Our work holds promise for personalized TNBC therapy and carries significant clinical translational potential. Although uncertainties exist during the antigen extraction process, the identified chemotherapy‐upregulated neoantigens exhibit comparable efficacy to tumor antigens derived from untreated cancer cells and could serve as potential substitutes. Furthermore, our strategy warrants further evaluation in more advanced animal models, such as humanized tumor mouse (HTM) models or investigator‐initiated trials (IITs).

However, several drawbacks of the current study should be acknowledged. First, beyond TAAs, neoantigens and DAMPs, our obtained chemo‐rAg also contains a considerable fraction of autoantigens, which may potentially impair the ultimate therapeutic efficacy of the prepared nanovaccine. Second, this cancer vaccine strategy has only been verified in murine tumor models as a proof of concept but lacks data on human BC models, which requires more exploration before its clinical translation. Accordingly, follow‐up investigations can be conducted in human TNBC cell line‐derived xenograft (CDX) models, as well as mouse model with a humanized immune system, to bridge the gap between concept validation and clinical application.

## 4 Experimental Section

### 4.1 Materials

Paclitaxel (PTX), carboplatin (CBP), 7‐allyl‐8‐oxoguanosine (loxoribine, loxo), and dichlorodiethylsilane were purchased from Shanghai Aladdin Biochemical Technology Co., Ltd. (Shanghai, China). 1,2‐Dioleoyl‐sn‐glycero‐3‐phosphoglycerol (DOPG), 1,2‐distearoyl‐sn‐glycero‐3‐phosphoethanolamine‐N‐[methoxy(polyethyleneglycol)‐2000] (DSPE‐mPEG_2000_) and cholesterol were purchased from Avanti Polar Lipids (Alabaster, AL, USA). Cyanine5.5 NHS ester (Cy5.5‐NHS) was purchased from Xi'an Ruixi Biological Technology Co., Ltd. (Xi'an, China). All chemicals were used as received without further purification.

Six neoantigen peptides, Actn2 (NIEEDFRKGLKLMLL), Dync1h1 (KPNYIVPDC), Foxn2 (RPKATFVGS), Fam111a (FDYKELLPT), Mga (EFGVSPRIG), and Nlrc5 (LSFHKFPLT), with a purity of >99%, were synthesized by GenScript Biotech Corporation (Nanjing, China). Modified Bradford protein assay kits were purchased from Sangon Biotech (Shanghai, China) Co., Ltd. Recombinant murine IL‐4 and granulocyte macrophage colony stimulatory factor (GM‐CSF) were obtained from Peprotech (Rocky Hill, NJ, USA), and anti‐PD‐L1 (clone: 10F.9G2) was purchased from BioXcell (Lebanon, NH, USA). Matrigel and fluorescence‐labeled anti‐mouse monoclonal antibodies (CD4, CD8a, CD80, CD86, and CD16/CD32 (Fc Block)) were obtained from BD Biosciences (San Jose, CA, USA), while other fluorescence‐labeled anti‐mouse monoclonal antibodies (B220, F4/80, CD45, CD3, Foxp3 and CD11c) were obtained from eBioscience (San Diego, CA, USA).

Anti‐CD3, anti‐CD4, and anti‐CD8 antibodies for IHC were purchased from Abcam (Cambridge, MA, USA). The live/dead fixable yellow dead cell stain kit and ACK lysis buffer were purchased from Life Technology (Carlsbad, CA, USA).

### 4.2 Prediction of Pre‐ and Post‐Chemotherapy Neoantigens in BC Patients

Pre‐ and post‐chemotherapy tumor samples were collected from eight BC patients (P1–P8) who received neo‐adjuvant chemotherapy (E represents epirubicin, C represents cyclophosphamide, T represents paclitaxel, H represents Herceptin, P represents pertuzumab), including a TNBC patient (P8). Treatment‐naive core‐needle biopsy samples were reserved as pre‐chemotherapy samples, while post‐chemotherapy tumor samples and adjacent normal tissue samples were collected from surgical specimens (Ethics Approval Number: 2025‐RE‐341).

Neoantigen identification was performed for both pre‐chemotherapy and post‐chemotherapy BC tumor samples. Briefly, whole‐exome sequencing (WES) was performed on both tumor and normal tissues, while RNA sequencing (RNA‐seq) and human leukocyte antigen (HLA) genotyping were carried out on tumor tissues and normal tissues, respectively. Using the WES data, non‐synonymous somatic mutations were first identified in the pre‐ and post‐chemotherapy samples. Later, RNA‐seq data were aligned to the human reference genome (hg19) and transcriptome (GENCODE v19) using Spliced Transcripts Alignment to a Reference (STAR, version 2.8.4a) [135] and expression was quantified in fragments per kilobase million (FPKM) using RNA‐Seq by Expectation‐Maximization (RSEM, version 1.3.1) [136]. All coding mutations were visualized using the Integrated Genomics Viewer (IGV). Thus, initial antigen candidates were generated. For each candidate, the corresponding 21‐mer peptide sequence centered on the mutation site and HLA genotype data (Data S6) were then subjected to affinity prediction using NetMHCPan‐4.1 and NetMHCIIpan‐4.1. Affinity for both HLA‐I and HLA‐II molecules was compared, ‘peptide score’ and ‘expression score’ were compared for the peptide candidates using a code that we reported [137, 138]. Based on these calculations, pre‐ and post‐chemotherapy neoantigens were identified.

Furthermore, RNA‐seq data of matched tumor tissues from BC patients were analyzed to evaluate the effects of chemotherapy on the TME. Specifically, immune cell abundance was calculated using the CIBERSORT algorithm, [139] while the tumor‐infiltrating immune cell scores were obtained through single‐sample gene set enrichment analysis (ssGSEA) [140].

### 4.3 Cell Lines and Animals

The 4T1, DC2.4 and RAW264.7 cell lines were all purchased from Cell Bank of Chinese Academy of Sciences (Shanghai, China) and cultured in Roswell Park Memorial Institute (RPMI) 1640 medium supplemented with 10% FBS (Gibco) and 1% penicillin‐streptomycin or Dulbecco's modified Eagle's medium (DMEM) containing 10% FBS, 1% GlutaMAX (Gibco), and 1% penicillin‐streptomycin, with 5% CO_2_ at 37°C.

BMDCs were prepared from bone marrow progenitor cells from BALB/c mice. Specifically, the bones were harvested, and marrow cells were flushed out with isolation medium using a needle. The cells were resuspended and seeded in 6‐well plates (2 × 10^6^ cells per well) with 2 mL RPMI‐1640 medium containing 10% FBS, 1% penicillin/streptomycin, 2 mM glutamine (Gibco), 20 ng mL^−1^ GM‐CSF, 10 ng mL^−1^ IL‐4, 55 µM β‐mercaptoethanol (β‐ME), and 10 mM HEPES. Cultures were maintained at 37°C with 5% CO_2_, and each well was supplemented with 20 ng mL^−1^ GM‐CSF and 10 ng mL^−1^ IL‐4. The medium was replaced every 2 days. Suspended and loosely adherent cells were collected on day 5 as immature BMDCs for subsequent experiments.

For all in vivo studies, 6–8‐week‐old female BALB/c mice were purchased from Shanghai Slac Laboratory Animal Co., Ltd. (Shanghai, China) and housed in specific‐pathogen free (SPF) rated environments with 12‐h light/dark cycles and provided with adequate food and water. Mice were used and randomly assigned to different treatment groups. Tumor volume was calculated using the formula: tumor volume = 0.5*length*width^2^. Mouse body weight and tumor volume were measured and recorded every 2–3 days. Mice were euthanized when tumor length exceeded 2 cm or when body weight decreased by more than 20%. All animal experiments were conducted in accordance with protocols approved by the China Experimental Animal Care Commission at the University of Science and Technology of China (no. USTCACUC25120124072) and complied with all relevant ethical regulations.

### 4.4 Establishment of PTX‐Resistant 4T1 Cell Line

The PTX‐resistant 4T1 cell line was established by gradually increasing the PTX concentration in the culture medium, starting at 1/10 of the half maximal inhibitory concentration (IC_50_) of the parental 4T1 cells. The resistance of PTX‐resistant 4T1 cells was validated by determining their IC_50_ value. Briefly, cells were seeded into 96‐well plates at a density of 3 × 10^3^ cells per well. The following day, the medium was replaced with fresh RPMI‐1640 medium containing varying concentrations of PTX. After 24 h of incubation, cell viability was assessed using the methylthiazolyldiphenyl‐tetrazolium bromide (MTT) assay. The IC_50_ values of PTX‐resistant and parental 4T1 cells were finally confirmed by MTT assay (Figure S27), yielding a resistance index of approximately 67 (4.644 µM/0.06854 µM).

### 4.5 Prediction of Tumor Neoantigens in Mice

Tumor neoantigen prediction in mice was similarly performed as described in BC patients. Female BALB/c mice of 6‐ to 8‐week‐old were subcutaneously implanted with 4T1 tumor cells. When tumors reached approximately 100 mm^3^, mice were intravenously injected with PBS or different chemotherapeutics (q3d, 4 doses). One day after the final administration, mice were euthanized, and tumor tissues were harvested and snap‐frozen in liquid nitrogen for subsequent analyses. Blood was collected from untreated BALB/c mice.

Then, neoantigen identification was performed for both pre‐chemotherapy and post‐chemotherapy mouse tumor samples. Briefly, WES was performed on both tumor and blood samples, while RNA‐seq was carried out on tumor tissues. Using the WES data, non‐synonymous somatic mutations were first identified in the pre‐ and post‐chemotherapy samples. Later, RNA‐seq data were aligned to the mus musculus reference genome (GRCm39) and transcriptome (GENCODE M39) using STAR (version 2.8.4a), and expression was quantified in FPKM using RSEM, (version 1.3.1). All coding mutations were visualized using the IGV. Thus, initial antigen candidates were generated. For each candidate, the corresponding 21‐mer peptide sequence centered on the mutation site was then subjected to affinity prediction using NetMHCPan‐4.1 and NetMHCIIpan‐4.1. Affinity for both MHC‐I (H‐2K^d^) and MHC‐II (I‐A^d^, I‐E^d^) molecules was compared. Based on these calculations, neoantigens in pre‐ and post‐chemotherapy mouse tumors were predicted.

### 4.6 Preparation of Tumor Antigens

PTX‐resistant 4T1 cells were treated with PTX, CBP or their combination (PTX + CBP) to generate tumor antigens derived from chemotherapy‐treated PTX‐resistant 4T1 cells (PTX‐rAg, CBP‐rAg and PTX/CBP‐rAg), respectively. Briefly, PTX‐resistant 4T1 cells were cultured to 80% confluence, washed three times with PBS, then incubated in FBS‐free RPMI‐1640 medium containing PTX (20 µM), CBP (120 µM) or PTX (20 µM) + CBP (120 µM) for 24–48 h. The culture supernatant and adherent cells were harvested, combined, and subjected to three freeze‐thaw cycles using liquid nitrogen. The mixture was centrifuged at 10, 000 g for 30 min at 4°C to remove insoluble cell debris. The resulting supernatant was concentrated using an Amicon stirred cell (3 kDa molecular weight cutoff (MWCO), Millipore, Burlington, MA, USA) and washed three times with cold PBS to eliminate residual small molecules (e.g., chemotherapeutics). As controls, non‐chemotherapy‐induced tumor antigens derived from parental 4T1 cells (Ag) and PTX‐resistant 4T1 cells (rAg) were extracted via direct freeze‐thawing, following the same protocol. Antigen concentrations were determined using a modified Bradford protein assay kits and then stored at −80°C until use.

To verify the absence of residual drugs in the chemotherapy‐induced tumor antigens, PTX (positive control, 0.01, 0.1, 1, 10, 20, 40, 60, 80, and 100 µg mL^−1^ in PBS containing 10% dimethyl sulfoxide (DMSO)), rAg (negative control, 4 mg mL^−1^ in 10% DMSO/PBS), and PTX‐rAg (4 mg mL^−1^ in 10% DMSO/PBS) were analyzed using a microplate reader (SpectraMax M3, Molecular Device, Sunnyvale, CA, USA). PTX exhibited a specific absorption peak at 246 nm, while the absorption spectrum of PTX‐rAg was nearly identical to that of rAg, with no observable increase at 246 nm (Figure S28), confirming minimal to no residual PTX in PTX‐rAg. Besides, high performance liquid chromatography‐mass spectrometry (HPLC‐MS) was also used to verify no residual PTX in PTX‐rAg (Figure S29).

### 4.7 Proteomic Analysis

The various tumor antigens samples collected above were subjected to mass spectrum (MS) analysis following reduction, alkylation, and trypsin digestion. Resulting peptides were dissolved in solvent A (0.1% formic acid, 2% acetonitrile in water) and directly loaded onto a homemade reversed‐phase analytical column (25 cm length, 75/100 µm i.d.). Peptide separation was performed using a gradient elution program on a nanoElute UHPLC system (Bruker Daltonics, Billerica, MA, USA) from 5% to 25% solvent B (0.1% formic acid in acetonitrile) at a constant flow rate of 450 nL/min over 50 min, 25% to 35% over 14 min, ramped to 80% over 3 min and held at 80% for a final 3 min. Peptides were introduced into a timsTOF Pro mass spectrometry (Bruker Daltonics, USA) via a capillary source with an applied electrospray voltage of 1.60 kV. Precursor and fragment were detected using the TOF detector with an MS/MS scan range of 100 to 1700 m/z. The instrument was operated in parallel accumulation serial fragmentation (PASEF) mode. Precursors with charge states of 0–5 were selected for fragmentation, with 10 PASEF‐MS/MS scans acquired per cycle. Dynamic exclusion was set to 30 s. Obtained MS/MS data were processed and searched against mouse entries of the SwissProt protein database (UniProtKB/Swiss‐Prot protein knowledgebase release 2021_07, Mouse taxonomy, 17,089 entries) using MaxQuant software (version 1.6.15.0). Trypsin/P was specified as a cleavage enzyme allowing up to 2 missing cleavages. The mass tolerance for precursor ions was set as 20 ppm in the first search and 5 ppm in the main search, and the mass tolerance for fragment ions was set as 0.02 Da. Carbamidomethyl on Cys was specified as a fixed modification, and acetylation on the protein N‐terminal and oxidation on Met were specified as variable modifications. The false discovery rate (FDR) was adjusted to <1%.

For neoantigen identification, a library of previously reported neoantigens was constructed [82, 83, 84] consisting of mutated amino acid sequences with 30 amino acids flanking both upstream and downstream of the mutation site (Data S4). Only detected peptide sequences covering the mutation sites were confirmed as neoantigens.

### 4.8 Preparation and Characterization of the Nanovaccines

The Lipo‐(Loxo+rAg) nanovaccine was self‐assembled using a standard film‐dispersion and hydration‐sonication method. DOPG, DSPE‐mPEG_2000_ and the as prepared chol‐loxo were dissolved in chloroform in a 10 mL round‐bottom glass flask at a mass ratio of 11.94 mg/8.34 mg/3.57 mg [46]. After solvent removal by rotary evaporation, the resulting thin lipid film was hydrated with 1 mL PBS (10 mM, pH = 7.2–7.4) containing 2.6 mg rAg and sonicated (60 W, 15 min). The turbid lipid suspension was extruded approximately 20 times through a 100 nm polycarbonate film using an Avanti Mini‐Extruder (Avanti Polar Lipids, Alabaster, AL, USA). Non‐encapsulated loxoribine and tumor antigens were removed by centrifugal elution using a 100 kDa MWCO ultrafiltration tube (Millipore, Burlington, MA, USA). Nanovaccines with different components (Lipo‐Loxo, Lipo‐(Loxo+PTX‐rAg), Lipo‐(Loxo+CBP‐rAg), Lipo‐(Loxo+PTX/CBP‐rAg) and Lipo‐(Loxo+Neo‐Ag)) were prepared following the same protocol.

The particle size distribution, PDI, and zeta potential of the prepared nanovaccine were measured using dynamic light scattering (Zetasizer Nano ZSE, Malvern, UK). The morphology of Lipo‐(Loxo+rAg) was observed by cryo‐transmission electron microscopy (Cryo‐TEM, Glacios, Thermo Fisher, USA). SDS‐PAGE was used to evaluate the antigen loading efficiency. The stability of Lipo‐(Loxo+rAg) under physiological conditions was investigated by monitoring changes in particle size and PDI in PBS or RPMI‐1640 complete medium (containing 10% FBS).

### 4.9 Loading and In Vitro Release of Antigens and Loxoribine

A standard curve for loxoribine was generated by measuring the absorption values of serially diluted solution (DMSO: water: hydrochloric acid = 40:9:1) with various concentrations at 299 nm using a microplate reader. Antigen quantification was performed using a modified Bradford protein assay kit.

To determine encapsulation efficiency and drug loading efficiency, the prepared nanovaccine Lipo‐(Loxo+rAg) was centrifuged using a 100 kDa MWCO ultrafiltration tube to separate free antigens and loxoribine from the nanovaccine. Free antigens were quantified using the modified Bradford assay, while free loxoribine was quantified based on the standard curve using the microplate reader (Table S1).

For release kinetics, 100 µL of Lipo‐(Loxo+rAg) solution (containing 1 mg mL^−1^ Ag and 0.5 mg mL^−1^ loxoribine) was added to 30 dialysis cups with a MWCO matched to retain the nanovaccine. Half of the cups were immersed in 4 L PBS (pH = 7.4), and the other half in 4L sodium acetate buffer (pH = 5.5). All systems were maintained under gentle shaking at 37°C. At predetermined time intervals (0.5, 1, 2, 3, and 4 days), three dialysis cups were removed to quantify residual content. Antigen concentrations were measured using the modified Bradford kit. For loxoribine quantification, the nanovaccine was demulsified by mixing with the solvent system (DMSO: water: hydrochloric acid = 40:9:1) to release encapsulated loxoribine, and absorbance was measured.

### 4.10 Cellular Uptake and Intracellular Localization of the Nanovaccine In Vitro

Bovine serum albumin (BSA) was incubated with Cy5.5‐NHS ester at a mass ratio of 100:1 for 4 h. Cy5.5‐labled BSA (Cy5.5‐BSA) was purified by removing excess Cy5.5‐NHS ester using a 3 kDa MWCO ultrafiltration tube. Cy5.5‐BSA was used as an antigen mimic to prepare Lipo‐(Loxo+Cy5.5‐BSA) following the protocol described above. Cellular uptake by DC2.4 cells was visualized using a confocal laser scanning microscope (CLSM, STELLARIS STED, Leica, Germany). Briefly, DC2.4 cells were seeded in confocal dishes (35 mm^2^, cover‐glass bottom) and incubated for 12 h at 37°C. Free‐(Loxo+Cy5.5‐BSA) or Lipo‐(Loxo+Cy5.5‐BSA) was added for co‐incubation with the cells for 4 h. After three washes with PBS, cells were stained with Hoechst 33342 at the optimal working concentration for 30 min. Following three additional PBS washes, cells were imaged using CLSM, and fluorescence intensity was quantified using ImageJ (version 1.54f).

For intracellular localization analysis, Lipo‐(Loxo+Cy5.5‐rAg) was prepared using the same method. DC2.4 cells were seeded in confocal dishes and then co‐incubated with Lipo‐(Loxo+Cy5.5‐rAg) for 4 h. ER‐Tracker Green and Lyso‐Tracker Green were used to stain ER and endosomes, respectively, and Hoechst 33342 was used for nuclear staining. Cells were washed three times with PBS and visualized by CLSM.

The cytotoxicity of Lipo‐(Loxo+rAg) to APCs was evaluated. DC2.4 and RAW264.7 cells were pre‐seeded into 96‐well plates (3 × 10^3^ per well) and then incubated with Lipo‐(Loxo+rAg) at different concentrations for 24 h. Cell viability was determined using the MTT assay. Untreated cells served as the control.

### 4.11 In Vitro Maturation of BMDCs

BMDCs were seeded into 6‐well plates at a density of 10^6^ cells per well and incubated with PBS, Free‐(Loxo+rAg), Lipo‐(Loxo+rAg), Lipo‐(Loxo+PTX‐rAg), Lipo‐(Loxo+CBP‐rAg) or Lipo‐(Loxo+PTX/CBP‐rAg) (100 µg mL^−1^ Ag and 50 µg mL^−1^ Loxo) for 24 h. Cells and culture supernatants were then collected. Prior to surface staining, BMDCs were incubated with Fc Block for 30 min on ice to block non‐specific antibody binding. Cells were stained with anti‐mouse CD11c, CD80, and CD86 antibodies, and the expression of the costimulatory molecules (CD80 and CD86) was analyzed by flow cytometry (CytoFLEX, Beckmann, Brea, CA, USA). Concentrations of IFN‐γ, TNF‐α, IL‐1β, IL‐6, and IL‐12 in BMDC supernatants were measured using ELISA kits (Ruixin Biotech, China) according to the manufactory's protocols.

### 4.12 In Vivo Uptake of the Nanovaccine by APCs in Draining Lymph Nodes

Healthy female BALB/c mice (6–8 weeks old) were subcutaneously injected into the flanks with Free‐(Loxo+Cy5.5‐rAg) or Lipo‐(Loxo+Cy5.5‐rAg). At 1 h and 24 h post‐injection, adjacent DLNs were harvested and imaged using an in vivo imaging system (IVIS, PerkinElmer, Waltham, MA, USA). Cy5.5 fluorescence intensity was quantified using Living Image Software (version 4.4). To assess the uptake of Free‐(Loxo+Cy5.5‐rAg) or Lipo‐(Loxo+Cy5.5‐rAg) by APCs in DLNs, DLNs were harvested and minced to prepare single‐cell suspensions. Cells were stained with anti‐mouse CD11c, F4/80, and B220 antibodies to identify APC subsets (DCs, macrophages, and B cells). Nanovaccine uptake by each subset was analyzed by flow cytometry.

### 4.13 In Vivo DC Maturation Induced by the Nanovaccine

Healthy female BALB/c mice (6–8 weeks old) were subcutaneously injected with PBS, Free‐(Loxo+rAg), Lipo‐(Loxo+rAg), Lipo‐(Loxo+PTX‐rAg), Lipo‐(Loxo+CBP‐rAg) or Lipo‐(Loxo+PTX/CBP‐rAg) (400 µg Ag and 200 µg Loxo per mouse). Mice were euthanized 24 h post‐treatment, and DLNs were harvested for analysis.

For flow cytometric analysis, DLNs were processed into single‐cell suspensions as described above and stained with anti‐mouse CD11c, CD80, and CD86 antibodies. For histological analysis, additional DLNs were fixed in 4% paraformaldehyde (PFA) for 24 h, embedded in paraffin, and sectioned into 5 µm slices. Immunofluorescence staining was performed using DAPI, anti‐CD80, and anti‐CD86 antibodies. Images were captured using a fluorescence microscope (Axiovert A1, Zeiss, Germany) and analyzed with ImageJ.

### 4.14 In Vivo Biosafety Analysis of the Nanovaccine

To evaluate in vivo biosafety, healthy female BALB/c mice were subcutaneously injected with 200 µl of PBS or 200 µl of Lipo‐(Loxo+PTX‐rAg) (400 µg PTX‐rAg and 200 µg Loxo per mouse) on days 0, 3, and 6. Mice were injected with PTX (5 mg kg^−1^) via the tail vein following the same schedule. Whole blood was collected from the retroorbital plexus 1 day after the first and final injections. Hematological parameters (HCT, MCV, RBC, HGB, WBC and PLT) were measured using an automated hematology analyzer. One week after the final injection, mice were euthanized. Serum was isolated by centrifugation, and biochemical parameters (ALT, AST, BUN, and CRE) were quantified using an automated chemistry analyzer. Heart, liver, spleen, lung and kidney tissues were harvested, fixed in 4% PFA, embedded in paraffin, and sectioned into 5 µm slices. Sections were stained with H&E and examined under a fluorescence microscope (Axiovert A1, Zeiss) for signs of tissue damage or inflammation.

### 4.15 In Vivo Antitumor Efficacy Studies of the Nanovaccine

The antitumor efficacy of the designed nanovaccines was evaluated in both normal 4T1 tumor‐bearing mice and PTX‐resistant 4T1 tumor‐bearing mice. To establish the murine 4T1 tumor model, 5 × 10^5^ normal 4T1 cells or PTX‐resistant 4T1 cells were suspended in 50 µL RPMI‐1640 medium, mixed with 50 µL matrigel, and subcutaneously injected into the right flank of female BALB/c mice (6–8 weeks old) on day 0. When tumor volume reached 50–100 mm^3^, mice were randomly assigned to different treatment groups.

To investigate the therapeutic advantage of the nanovaccine, tumor‐bearing mice received subcutaneous injections of different treatments into the left flank on days 3, 6, and 9 post‐tumor inoculation, including PBS (negative control), Free‐(Loxo+PTX‐Ag) and Lipo‐(Loxo+PTX‐Ag) (20 mg kg^−1^ Ag and 4 mg kg^−1^ Loxo).

To verify the efficacy in the PTX‐resistant tumor model and assess the synergistic effect of ICIs with the nanovaccine, Free‐(Loxo+PTX‐rAg) or Lipo‐(Loxo+PTX‐rAg) (20 mg kg^−1^ Ag and 10 mg kg^−1^ Loxo, s.c.) were injected into the left flank of tumor‐bearing mice on days 6, 9, and 12 post‐tumor inoculation. Anti‐PD‐L1 antibody (5 mg kg^−1^, i.p.) was administered on the same schedule.

To evaluate the advantages of chemotherapy‐induced tumor antigens (PTX‐rAg, CBP‐rAg and PTX/CBP‐rAg) over untreated tumor antigens (rAg), PTX‐resistant 4T1 tumor‐bearing mice were used. Lipo‐(Loxo+rAg), Lipo‐(Loxo+PTX‐rAg), Lipo‐(Loxo+CBP‐rAg) or Lipo‐(Loxo+PTX/CBP‐rAg) (20 mg kg^−1^ Ag and 10 mg kg^−1^ Loxo, s.c.) were injected into the left flank of the mice on days 5, 8, and 11. Anti‐PD‐L1 antibody (5 mg kg^−1^, i.p.) was administered on the same schedule.

To explore the efficacy of chemotherapy‐induced in situ tumor antigens, PTX (5 mg kg^−1^) was intravenously (i.v.) injected on day 5. On days 8 and 11, mice received Lipo‐(Loxo+PTX‐rAg) (20 mg kg^−1^ Ag and 10 mg kg^−1^ Loxo, s.c.) injections, with anti‐PD‐L1 (5 mg kg^−1^, i.p.) administered on days 5, 8, and 11 post‐tumor inoculation.

Neoantigens are well‐documented to possess significant advantages in inducing antitumor immune response [114, 141, 142, 143]. To evaluate the liposome nanovaccine based on neoantigens, Lipo‐(Loxo+Neo‐Ag) was prepared using two PTX‐upregulated neoantigens (Actn2 and Dync1h1) and compared with Lipo‐(Loxo+PTX‐rAg) in the PTX‐resistant 4T1 tumor model. PTX (5 mg kg^−1^, i.v.) was injected on day 5. On days 5, 8, and 11 post‐tumor inoculation, mice were vaccinated with Lipo‐(Loxo+PTX‐rAg) or Lipo‐(Loxo+Neo‐Ag) (10 mg kg^−1^ Ag and 10 mg kg^−1^ Loxo, s.c.), with concurrent administration of anti‐PD‐L1 (5 mg kg^−1^, i.p.).

To test the efficacy of the predicted neoantigens, another neoantigen nanovaccine Neo‐VAX was prepared with 4 predicted neoantigen peptides (Foxn2, Fam111a, Mga, and Nlrc5) through the similar method. The antitumor effect of Neo‐VAX was investigated in the PTX‐resistant 4T1 tumor model. On days 6, 9, and 12 post‐tumor inoculation, mice were vaccinated with Neo‐VAX (10 mg kg^−1^ Ag and 10 mg kg^−1^ Loxo, s.c.), with concurrent administration of anti‐PD‐L1 (5 mg kg^−1^, i.p.).

### 4.16 Analysis of Tumor‐Infiltrating T‐Cells

One week after the final treatment, mice were euthanized, and tumors were harvested. Tumors were processed into single‐cell suspensions for flow cytometry analysis. Cells were stained with anti‐mouse CD45, CD3, CD4, and CD8 antibodies. Following surface staining, cells were fixed, permeabilized, and stained intracellularly with anti‐mouse Foxp3 antibody. Samples were analyzed using a CytoFLEX flow cytometer (Beckman Coulter, Brea, CA, USA) with CytExpert (version 2.3).

### 4.17 Antitumor Immune Memory Effect of the Nanovaccine

For tumor rechallenge studies, cured mice that had rejected primary tumors following combination treatment with the nanovaccine and anti‐PD‐L1 (and remained tumor‐free for at least 90 days) were subcutaneously implanted with 5 × 10^5^ PTX‐resistant 4T1 cells into the left flank. Naïve mice (female BALB/c, 6–8 weeks old) implanted with 5 × 10^5^ PTX‐resistant 4T1 cells served as controls.

### 4.18 Adoptive Transfer Therapy

To further investigate the immune memory effect of the nanovaccines, cured mice that had rejected the tumors following combination treatment with the nanovaccine and anti‐PD‐L1 were used as the source of splenocytes. Cured mice were euthanized, and their spleens were harvested to prepare single‐cell suspensions. Naive mice (8–10 weeks old) were intravenously injected with 200 µL cold PBS containing 4 ×10^6^ splenocytes from cured mice or untreated mice. Naïve mice injected with 200 µL PBS (i.v.) served as an additional control group. Two days later, each group was subcutaneously implanted with 5 × 10^5^ PTX‐resistant 4T1 cells. Tumor volumes and body weights were monitored every 2–3 days for 20 days. IHC staining for CD3, Foxp3, CD4, and CD8 was then performed to analyze Treg, CD4^+^, and CD8^+^ T cell infiltration in tumor tissues.

### 4.19 IHC Analysis of Tumor Tissues

At the end of the antitumor efficacy experiment, mice were euthanized, and tumors from different treatment groups were collected. Tumor tissues were fixed with 4% PFA at 4°C, embedded in paraffin, and sectioned into 5‐µm slices for analysis. Tissue sections were stained with H&E, and images were captured using a bright field microscope (Axiovert A1, Zeiss, Germany). For IHC staining, tumor sections were incubated with anti‐mouse CD3, Foxp3, CD4, or CD8 primary antibodies, followed by horseradish peroxidase‐conjugated IgG secondary antibody, according to the manufacturer's protocols. After treatment with diaminobenzidine, sections were imaged under the microscope (Axiovert A1, Zeiss, Germany).

### 4.20 Ex Vivo T‐Cell Proliferation Assay

Spleens were harvested from naïve mice to prepare single‐cell suspensions. Splenocytes were resuspended in PBS containing 5 µM CFSE to a density of 5 × 10^6^ cells mL^−1^ and stained for 10 min at room temperature in the dark. After two washes with PBS, 5 × 10^5^ CFSE‐labeled splenocytes were added to RPMI‐1640 complete medium and co‐incubated with 2 × 10^5^ BMDCs pre‐stimulated with different antigens (100 µg mL^−1^). Two days later, cells were collected, washed with PBS, and stained with anti‐mouse CD3, CD4, and CD8 antibodies. Samples were analyzed using a CytoFLEX flow cytometer (Beckman Coulter, USA) with CytExpert (version 2.3) to assess T‐cell proliferation across treatments.

### 4.21 Ex Vivo IFN‐γ Secretion via Flow Cytometry

On day 0, 5 × 10^5^ PTX‐resistant 4T1 cells were subcutaneously injected into the right flank of female BALB/c mice. When tumor volumes reached 50–100 mm^3^, mice were randomly assigned to seven treatment groups: (1) PBS, (2) anti‐PD‐L1, (3) Lipo‐(Loxo+rAg) + anti‐PD‐L1, (4) Lipo‐(Loxo+PTX‐rAg) + anti‐PD‐L1, (5) Lipo‐(Loxo+Neo‐Ag) + anti‐PD‐L1, (6) PTX + Lipo‐(Loxo+PTX‐rAg) + anti‐PD‐L1, (7) PTX + Lipo‐(Loxo+Neo‐Ag) + anti‐PD‐L1. On days 5, 8, and 11, nanovaccines were subcutaneously injected into the left flanks, and anti‐PD‐L1 antibody was administered intraperitoneally.

One week after the final treatment, splenocytes were harvested and cultured in RPMI 1640 complete medium containing nonessential amino acids (NEAAs) and IL‐2 (10 ng mL^−1^). The cells were seeded into 12‐well plates (10^6^ cells per well) and restimulated with different antigens (100 µg mL^−1^). Brefeldin A and monensin (Multi Scinences, China) were added to block the IFN‐γ transport outside of the cells. Phorbol‐12‐myristate‐13‐acetate and ionomycin (Multi Scinences, China) were added as positive control groups. After 72 h, splenocytes were incubated with a live/dead fixable yellow death cell stain kit (Invitrogen, USA) and Fc block. Next, anti‐mouse CD3, CD4 and CD8 antibodies were used for surface staining at 4°C for 30 min. Cells were then fixed, permeabilized, and stained with anti‐mouse IFN‐γ antibody. The relative abundance of IFN‐γ^+^ CD8^+^ T cells and IFN‐γ^+^ CD4^+^ T cells was detected by Beckmann flow cytometer (CytoFLEX).

### 4.22 TME RNA Sequencing in Mice

The mice were divided into seven groups: PBS, anti‐PD‐L1, Lipo‐(Loxo+rAg) + anti‐PD‐L1, Lipo‐(Loxo+PTX‐rAg) + anti‐PD‐L1, Lipo‐(Loxo+Neo‐Ag) + anti‐PD‐L1, PTX + Lipo‐(Loxo+PTX‐rAg) + anti‐PD‐L1, PTX + Lipo‐(Loxo+Neo‐Ag) + anti‐PD‐L1. On days 5, 8, and 11 post‐tumor inoculation, the mice received the specified treatments as outlined. The dosing regimen followed the method detailed in the main text. One week after the final treatment, tumors were rapidly excised, washed with pre‐cooled PBS, and immediately frozen in liquid nitrogen to prevent the degradation of RNA.

Subsequently, tumor samples were sent to Biozeron Biotechnology Co., Ltd. (Shanghai, China) to perform RNA‐seq and transcriptome library construction. The method for creating a library from tumor tissue involved several steps. First, tumor tissues were minced, and RNA was extracted using TRIzol reagent. After rRNA removal using a standard kit, mRNA was enriched. The quality of the mRNA was assessed by evaluating its integrity, purity, and concentration. Once the mRNA passed quality control, the Illumina TruSeq RNA Sample Preparation Kit was used for library construction. This involved enriching poly‐A‐tailed mRNA using magnetic beads coated with Oligo(dT). The mRNA was then fragmented by sonication to create short RNA fragments. The first cDNA strand was synthesized using M‐MuLV reverse transcriptase, followed by RNA degradation with RNase H and second‐strand cDNA synthesis using DNA polymerase I and dNTPs. The double‐stranded cDNA was end‐repaired, adenylated, and sequencing adapters were ligated. AMPure XP beads were used to select cDNA around 200 bp in size, and PCR amplification was performed to generate the library. The library was then purified using AMPure XP beads.

After passing quality control, the libraries underwent next‐generation sequencing. The sequencing results were aligned and annotated against the reference genome using Hisat2 software. Subsequent analyses, including differential expression, functional annotation, and functional enrichment (Gene Set Enrichment Analysis, GSEA), were performed for each group. Immune cell abundance was analyzed using the ImmuCellAI‐mouse algorithm [94].

### 4.23 Statistical Analysis

In this study, the sample size was not pre‐determined. All data results from parallel samples are presented as the mean ± standard error of the mean (SEM), and statistical analyses were conducted using GraphPad Prism (version 8). Quantification and colocalization analysis of the fluorescence images were performed using ImageJ (version 1.54f), while all flow cytometry data were acquired on a CytoFLEX Flow Cytometer (Beckman) and analyzed using CytExpert (version 2.3) or FlowJo (version 10.6).

In animal experiments, mice were randomly assigned into different treatment groups, and the experiments were single‐blinded. Data analyses were also single‐blinded, including RNA‐Seq data analysis. All in vitro studies were not blinded, as results were obtained through objective measurements. No data were excluded from the analysis. Statistical comparisons between groups were performed using unpaired t‐test or one‐way analysis of variance (ANOVA). Two‐way ANOVA was used to compare tumor volume curves over time between different groups. For survival analysis, Kaplan‐Meier curves were generated, and the log‐rank test was used to determine the overall *p*‐value.

## Author Contributions

Y.M. conceived and designed the experiments with B.C. B.C. conducted both in vitro and in vivo experiments with the help of Y.W. Y.W and X.M. collected samples from clinic and processed the related data. T.L. and J.W. processed data of tumor neoantigens. W.W help with the bioinformatics data. X.M., W.W., and X.H. are in charge of clinical related matters and the related design of efficacy study on mice. Y.M., B.C., and Y.W. wrote the manuscript. All authors analyzed, discussed the data and approved the submission.

## Funding

This work was funded by National Nature Science Foundation of China (31971299) for Y.M. A startup grant from Anhui Provincial Hospital (RC2021117) and Health Research Program of Anhui (AHWJ2025BAq40017) for Y.W. The Strategic Priority Research Program, the Chinese Academy of Sciences (XDB0940303), the Institute of Health and Medicine, Hefei Comprehensive National Science Center (DJK‐LX‐2022005), the Natural Science Foundation of China (82271827, 32470954) for J.W.

## Conflicts of Interest

The authors declare no conflicts of interest.

## Supporting information




**Supporting File 1**: advs77249‐sup‐0001‐SuppMat.docx.

**Supporting File 2**: advs77249‐sup‐0002‐S1.xlsx.

**Supporting File 3**: advs77249‐sup‐0003‐S2.xlsx.

**Supporting File 4**: advs77249‐sup‐0004‐S3.xlsx.

**Supporting File 5**: advs77249‐sup‐0005‐S4.xlsx.

**Supporting File 6**: advs77249‐sup‐0006‐S5.xlsx.

**Supporting File 7**: advs77249‐sup‐0007‐S6.csv.

## Data Availability

All data needed to evaluate the conclusions in this paper are available in the main text or the supplementary materials. The raw and analyzed datasets generated during the study are available for academic purposes from the corresponding authors upon request.
