# Supplementary material for: Overcoming Drug Resistance by Paclitaxel Resistance in Triple‐Negative Breast Cancer
Source: Adv Sci (Weinh). 2026 Aug 20:e77249. Online ahead of print. doi: 10.1002/advs.77249 (PMC13491373; doi:10.1002/advs.77249)
Supplement: Supplementary file 1 [file ADVS-9999-e77249-s004.docx]

Supporting Information

For

Overcoming Drug Resistance by Paclitaxel Resistance in Triple-Negative Breast Cancer

Bo Chen^1,#^, Yangyi Wang^1,#^, Tingting Li^3,#^, Changqing Guo^2^, Zhe Xie^1^, Zhixian Zhang^2^, Xinghua Han^1,*^, Wei Wang^1,*^, Jian Wang^1,*^, Xiaopeng Ma^1,*^, Yuanzeng Min^1,2,*^

^1^Department of General Surgery, Department of Medical Oncology, The First Affiliated Hospital of USTC, Division of Life Sciences and Medicine, University of Science and Technology of China, Hefei, China; Department of Bio-X Interdisciplinary Science at Hefei National Laboratory (HFNL) for Physical Science at the Microscale, University of Science and Technology of China, Hefei, China.

^2^School of Chemistry and Materials Science, University of Science and Technology of China, Hefei, China.

^3^Department of Clinical Laboratory, The First Affiliated Hospital of Anhui Medical University, Hefei, China.

^#^Bo Chen, Yangyi Wang and Tingting Li contributed equally to this work.

***Corresponding Author(s)**: Xinghua Han ([hxhmail@hotmail.com](mailto:hxhmail@hotmail.com)); Wei Wang ([docweiwang@ustc.edu.cn](mailto:docweiwang@ustc.edu.cn)); Jian Wang (ustcwj@ustc.edu.cn); Xiaopeng Ma ([xiaopengma@fsyy.ustc.edu.cn](mailto:xiaopengma@fsyy.ustc.edu.cn)); Yuanzeng Min (minyz@ustc.edu.cn).

**This PDF file includes:**

Figure S1-S29.

Table S1-S4.

**Other Supplementary Materials for this manuscript include the following:**

Data S1-S6.

**Figure S1.** The detailed information of the acid-responsive TLR7 agonist prodrug chol-loxo. (A) Schematic illustration showing the preparation of chol-loxo. (B) The high-resolution ESI mass spectrum of chol-loxo with a molecular formula of C_44_H_71_O_7_N_5_Si: [M + H] ^+^ calculated for C_44_H_72_O_7_N_5_Si, 810.51955; found, 810.52027. (C) ^1^H-NMR (400 MHz, Chloroform-d, δ) spectrum of chol-loxo: 10.92 (s, 1H), 8.01 (s, 1H), 7.71 (ddd, J = 5.7, 3.9, 3.3 Hz, 2H), 7.52 (ddd, J = 5.7, 3.3, 0.7 Hz, 2H), 5.40-5.30 (m, 1H), 4.30 (t, J = 6.7 Hz, 3H), 4.27-4.15 (m, 2H), 3.70-3.62 (m, 4H), 3.10 (qd, J = 7.4, 4.0 Hz, 2H), 2.95 (d, J = 0.5 Hz, 3H), 2.88 (d, J = 0.6 Hz, 3H), 2.34-2.27 (m, 1H), 2.25-2.16 (m, 1H), 2.01 (d, J = 7.3 Hz, 1H), 1.76-1.66 (m, 5H), 1.57-1.51 (m, 12H), 1.45 (t, J = 7.1 Hz, 11H), 0.92-0.85 (m, 12H), 0.08 (d, J = 3.3 Hz, 4H).

**Figure S2.** Venn diagram showing the predicted neoantigens in tumors from 8 BC patients (P1-P8) before and after chemotherapy (E represents epirubicin, C represents cyclophosphamide, T represents paclitaxel, H represents Herceptin, P represents pertuzumab), including 1 TNBC patient (P8).

**Figure S3.** KEGG analysis of differentially enriched genes in biological processes related to the immune response. For each patient, RNA-seq analysis was performed on tumor samples before and after chemotherapy. Analysis results corresponding to (A) patients P1-P4 and (B) patients P5-P8.

**Figure S4.** Proportion of different tumor-infiltrating immune cells in the tumor. For each patient, analysis was performed on tumor samples before and after chemotherapy. Analysis results corresponding to (A) patients P1-P4 and (B) patients P5-P8.

**Figure S5.** Heatmap of tumor-infiltrating immune cell scores *via* single-sample gene set enrichment analysis (ssGSEA). For each patient, analysis was performed on tumor samples before and after chemotherapy. Analysis results corresponding to (A) patients P1-P4 and (B) patients P5-P8.

**Figure S6.** Heatmap showing the relative expression levels of partial immune-related genes in the tumor. Analysis was performed on tumor samples from patients before and after chemotherapy. Analysis results corresponding to (A) patients P1-P4 and (B) patients P5-P8.

**Figure S7.** Heatmap showing the relative expression levels of partial immune-related genes in the tumor. Analysis was performed on tumor samples from patients before and after chemotherapy. Analysis results corresponding to (A) patients P1-P4 and (B) patients P5-P8.

**Figure S8.** Ultrasound examination results of 8 BC patients (P1-P8). Ultrasound examinations were performed on each patient before and after neoadjuvant chemotherapy.

**Figure S9.** Quantitative analysis of antigen uptake by DC2.4 cells. After incubating DC2.4 cells with Free-(Loxo+Cy5.5-BSA) or Lipo-(Loxo+Cy5.5-BSA) for 4 hours, the antigen uptake by DC2.4 cells was calculated by measuring mean fluorescence intensity of Cy5.5 (*n* = 3). Data are presented as the mean ± SEM. Statistical significance was calculated by unpaired t-test (**p* < 0.05, ***p* < 0.01, ****p* < 0.001, *****p* < 0.0001).

**Figure S10.** The cytotoxicity of the nanovaccine in vitro. After incubating Lipo-(Loxo+rAg) with DC2.4 or RAW264.7 cells for 24 hours, the cytotoxicity of Lipo-(Loxo+rAg) was assessed *via* the MTT assay (*n* = 3). Data are presented as the mean ± SEM.

**Figure S11.** The gating strategy of flow cytometric analysis corresponding to the BMDC maturation experiment (Figure 3C).

**Figure S12.** The gating strategy of flow cytometric analysis corresponding to the antigen uptake experiment in the DLNs (Figure 3G-3H).

**Figure S13.** The gating strategy of flow cytometric analysis corresponding to the DC maturation experiment in the DLNs (Figure 3J).

**Figure S14.** Antitumor effects of the nanovaccine in the non-resistant 4T1 tumor model. (A) Body weight change curves and (B) individual tumor growth curves of non-resistant 4T1 tumor-bearing BALB/c mice following different treatments in Figure 5A (*n* = 7). Data are presented as the mean ± SEM.

**Figure S15.** Antitumor effects of the nanovaccine in the PTX-resistant 4T1 tumor model. (A) Body weight change curves and (B) individual tumor growth curves of PTX-resistant 4T1 tumor-bearing BALB/c mice following different treatments in Figure 5B (*n* = 7-8). Data are all presented as the mean ± SEM.

**Figure S16.** Antitumor effects of different nanovaccines in the PTX-resistant 4T1 tumor model. (A) Body weight change curves and (B) individual tumor growth curves of PTX-resistant 4T1 tumor-bearing BALB/c mice following different treatments in Figure 5C (*n* = 6-7). Data are presented as the mean ± SEM.

**Figure S17.** Antitumor effects of PTX in the non-resistant 4T1 tumor model. (A) Schematic illustration of the treatment schedule (*n* = 7-8). (B) Average tumor growth curves and (C) survival curves of the tumor-bearing mice treated with PBS or PTX. (D) Body weight change curves and (E) individual tumor growth curves of the 4T1 tumor-bearing BALB/c mice following different treatments. Data are presented as the mean ± SEM. Tumor growth curves were analyzed by two-way ANOVA with Geisser-Greenhouse correction. Statistical differences of survival time were calculated by Kaplan Meier method and the p value was calculated by Gehan-Breslow-Wilcoxon test (**p* < 0.05, ***p* < 0.01, ****p* < 0.001, *****p* < 0.0001).

**Figure S18.** Antitumor effects of PTX and nanovaccine in the PTX-resistant 4T1 tumor model. (A) Body weight change curves and (B) individual tumor growth curves of the PTX-resistant 4T1 tumor-bearing BALB/c mice following different treatments in Figure 5D (*n* = 7-8). Data are presented as the mean ± SEM.

**Figure S19.** Gene Ontology (GO) analysis of differentially enriched genes in the upstream stress pathways related to the antigen repertoire changes. For PTX-resistant 4T1 cells or PTX-resistant 4T1 tumors, RNA sequencing was performed on corresponding samples before and after chemotherapy treatment.

**Figure S20.** Antitumor effects of neoantigen-based nanovaccine in the PTX-resistant 4T1 tumor model. (A) Body weight change curves and (B) individual tumor growth curves of the PTX-resistant 4T1 tumor-bearing BALB/c mice following different treatments in Figure 6F (*n* = 8). Data are presented as the mean ± SEM.

**Figure S21.** Antitumor effect of the neoantigen nanovaccine (Neo-VAX) in the PTX-resistant 4T1 tumor model (*n* = 6-7). Based on 4 predicted neoantigens (Foxn2, Fam111a, Mga and Nlrc5), Neo-VAX was prepared. (A) Schematic illustration of the treatment schedule. (B) Average tumor growth curves, (C) survival curves and (D) individual tumor growth curves of the tumor-bearing mice following different treatments. Data are presented as the mean ± SEM. Tumor growth curves were analyzed by two-way ANOVA with Geisser-Greenhouse correction. Statistical differences of survival time were calculated by Kaplan Meier method and the p value was calculated by Gehan-Breslow-Wilcoxon test (**p* < 0.05, ***p* < 0.01, ****p* < 0.001, *****p* < 0.0001).

**Figure S22.** The gating strategy of flow cytometric analysis corresponding to the ex vivo T-cell proliferation experiment (Figure 7A-7C).

**Figure S23.** The gating strategy of flow cytometric analysis corresponding to ex vivo IFN-γ secretion experiment (Figure 7D-7E).

**Figure S24.** RNA-seq analysis of mice tumors after different treatments. One week after the last treatment, tumor tissues from different treatment groups were collected for subsequent RNA-seq analysis (*n* = 3). (A) Heatmap showing the activation of representative immune-related pathways as determined by gene set enrichment analysis (GSEA). (B) Heatmap showing the expression levels of representative immune-related genes. (C-E) Immune cell infiltration in the TME was evaluated using the ESTIMATE (Estimation of Stromal and Immune Cells in Malignant Tumor Tissues Using Expression Data) algorithm, generating (C) tumor purity, (D) immune score, and (E) ESTIMATE score. (F-Z) Immune cell infiltration was further quantified using the Immune Cell Abundance Identifier for Mouse (ImmuCellAI-mouse) algorithm, generating (F) infiltration score, which reflected the relative abundance of immune cells in the TME across different groups. The relative abundance (RA) of intra-tumoral (G) DCs, (H) T cells, (I) Treg cells, (J) pDCs, (K) MoDCs, (L) NKT cells, (M) macrophages, (N) B cells, (O) memory B cells, (P) plasma cells, (Q) CD4^+^ T cells, (R) naïve CD4^+^ T cells, (S) CD4^+^ Tm cells, (T) CD8^+^ T cells, (U) naïve CD8^+^ T cells, (V) CD8^+^ Tc cells, (W) CD8^+^ Tcm cells, (X) CD8^+^ Tem cells, (Y) T helper cells and (Z) γδ T cells in each group was shown. Statistical significance was calculated by unpaired t-test (**p* < 0.05, ***p* < 0.01, ****p* < 0.001, *****p* < 0.0001).

**Figure S25.** Long-term antitumor immune memory induced by the nanovaccine. (A) Body weight change curves and (B) individual tumor growth curves of the cured or naïve mice after inoculate with PTX-resistant 4T1 tumors in Figure 8A (*n* = 4-5). Data are presented as the mean ± SEM.

**Figure S26.** Antitumor effects of adoptive splenocyte transfer therapy in the PTX-resistant 4T1 tumor model. (A) Body weight change curves and (B) individual tumor growth curves of the cured or naïve mice after inoculating with PTX-resistant 4T1 tumors in Figure 8D (*n* = 7-8). Data are presented as the mean ± SEM.

**Figure S27.** MTT assay to measure the IC_50_ values of non-resistant and PTX-resistant 4T1 cell lines. Data are presented as the mean ± SEM (*n* = 4).

**Figure S28.** Detection of PTX residue in the obtained PTX-rAg. (A) UV absorption spectra of standard PTX solutions prepared by dissolving PTX at different concentrations in a of PBS and DMSO (volume ratio of 9:1). (B) UV absorption spectra of obtained PTX-rAg, while rAg used as the negative control.

**Figure S29.** The detection of chemodrug residuals in PTX-treated 4T1 cell lysates. High performance liquid chromatography-mass spectrometry (HPLC-MS) analysis results of (A) the PTX standard solution (corresponding mass spectrometry peak of PTX : [M+Na]^+^, calculated at 876.3202, detected at 876.3500) and (B) the PTX-rAg solution.

**Table S1.** Encapsulation efficiency and loading efficiency of antigen or loxoribine in the liposome nanovaccine.

| Drugs | Encapsulation efficiency | Loading efficiency |
| --- | --- | --- |
| Antigen  Loxoribine | 68.12%  78.42% | 26.63%  15.33% |

The solution of Lipo-(Loxo+rAg) was centrifuged in an ultrafiltration tube (pore size: 100 kDa MWCO) to remove non-encapsulated free antigens and free loxoribine, which was quantified using a microplate reader. In this experiment, liposome components (19.950 mg), antigens (rAg, 7.800 mg) and chol-loxo (equivalent to 3.900 mg loxoribine) were mixed to prepare Lipo-(Loxo+rAg). After ultrafiltration, the non-encapsulated antigens and loxoribine were measured to be 2.487 mg and 0.8414 mg, respectively.

Encapsulation efficiency of antigens = (W_Total Ag_ - W_nonencapsulated Ag_) / W_Total Ag_

= (7.800 mg – 2.487 mg) / 7.800 mg

= 68.12%

Loading efficiency of antigens = (W_Total Ag_ - W_nonencapsulated Ag_) / W_Lipo_

= (7.800 mg – 2.487 mg) / 19.950 mg

= 26.63%

Encapsulation efficiency of loxoribine = (W_Total loxoribine_ - W_nonencapsulated loxoribine_) / W_Total loxoribine_

= (3.900 mg – 0.8414 mg) / 3.900 mg

= 78.42%

Loading efficiency of loxoribine = (W_Total loxoribine_ - W_nonencapsulated loxoribine_) / W_Lipo_

= (3.900 mg – 0.8414 mg) / 19.950 mg

= 15.33%

**Table S2.** Information on the detected DAMPs in tumor antigens derived from PTX-resistant 4T1 cancer cells after receiving different treatments in vitro.

| Gene name | Protein name |
| --- | --- |
| Triobp  Il4r  Hspa4l  Hdgf  Ssbp1  Prdx1  Ppia  Macroh2a1  Il6st  Hspa8  Hspa4  Hspa1b  Hspa13  Hsp90ab1  Hmgn1  Hmgb1  H1-4  H1-2  Carmil1  Capzb  Capza2  Bcl2l13 | TRIO and F-actin-binding protein  Interleukin-4 receptor subunit alpha  Heat shock 70 kDa protein 4L  Hepatoma-derived growth factor  Single-stranded DNA-binding protein, mitochondrial  Peroxiredoxin-1  Peptidyl-prolyl cis-trans isomerase A  Core histone macro-H2A.1  Interleukin-6 receptor subunit beta  Heat shock cognate 71 kDa protein  Heat shock 70 kDa protein 4  Heat shock 70 kDa protein 1B  Heat shock 70 kDa protein 13  Heat shock protein HSP 90-beta  Non-histone chromosomal protein HMG-14  High mobility group protein B1  Histone H1.4  Histone H1.2  F-actin-uncapping protein LRRC16A  F-actin-capping protein subunit beta  F-actin-capping protein subunit alpha-2  Bcl-2-like protein 13 |

**Table S3.** Information on the detected Neoantigens in tumor antigens derived from PTX-resistant 4T1 cancer cells after receiving different treatments in vitro.

| Gene | Protein | Sequence* |
| --- | --- | --- |
| Dync1h1  Actn2  Qars  Ilkap | Cytoplasmic dynein 1 heavy chain 1  Alpha-actinin-2  Glutamine-tRNA ligase  Integrin-linked kinase-associated serine/threonine phosphatase 2C | KPNYIVPD[Y_C]  NIEEDFR[N_K]GLKLMLL  FTLTALRRRGFPP[E_D]AINNFCARVGVTV  RKGEREEMQDAHV[I_S]LNDITQECNPPSS |

*: [Y_C] represent that the amino acid at the site is mutated from Y to C. These sequences were obtained from mass spectrometry results in proteomic analysis.

**Table S4.** Antibody information for flow cytometric analysis, IHC and immunofluorescence.

| Antibody | Fluorophore | Clone | Lot number | Vendor |
| --- | --- | --- | --- | --- |
| CD45  CD3  CD4  CD8α  Foxp3  IFN-γ  CD11c  B220  F4/80  CD80  CD86  CD3  CD4  CD8  Foxp3  CD11c  CD80  CD86 | eFluor® 450  APC-eFluor® 780  Alexa Fluor® 488  APC  PE-eFluor® 610  BV421  PE-Cyanine7  eFluor® 450  PE-eFluor® 610  BV421  APC  /  /  /  /  /  /  / | 30-F11  17A2  RM4-5  53-6.7  FJK-16s  XMG1.2  N418  RA3-6B2  BM8  16-10A1  GL1  ab16669  ab183685  98941T  12653T  GB11059  A16039  19589S | 48-0451-82  47-0032-82  557667  553035  61-5773-82  /  25-0114-82  48-0452-82  61-4801-82  562611  558703  GR3376661-11  GR3375645-1  2  9  AC221123047  3516304101  5 | BD Biosciences  BD Biosciences  BD Biosciences  BD Biosciences  BD Biosciences  BD Biosciences  BD Biosciences  BD Biosciences  BD Biosciences  BD Biosciences  BD Biosciences  Abcam  Abcam  CST  CST  Servicebio  Abclonal  CST |
